# Arabidopsis MDA1, a Nuclear-Encoded Protein, Functions in Chloroplast Development and Abiotic Stress Responses

**DOI:** 10.1371/journal.pone.0042924

**Published:** 2012-08-08

**Authors:** Pedro Robles, José Luis Micol, Víctor Quesada

**Affiliations:** Instituto de Bioingeniería, Universidad Miguel Hernández, Campus de Elche, Elche, Spain; Instituto de Biología Molecular y Celular de Plantas, Spain

## Abstract

Most chloroplast and mitochondrial proteins are encoded by nuclear genes, whose functions remain largely unknown because mutant alleles are lacking. A reverse genetics screen for mutations affecting the mitochondrial transcription termination factor (mTERF) family in *Arabidopsis thaliana* allowed us to identify 75 lines carrying T-DNA insertions. Two of them were homozygous for insertions in the At4g14605 gene, which we dubbed *MDA1* (*MTERF DEFECTIVE IN Arabidopsis1*). The *mda1* mutants exhibited altered chloroplast morphology and plant growth, and reduced pigmentation of cotyledons, leaves, stems and sepals. The *mda1* mutations enhanced salt and osmotic stress tolerance and altered sugar responses during seedling establishment, possibly as a result of reduced ABA sensitivity. Loss of *MDA1* function caused up-regulation of the *RpoTp/SCA3* nuclear gene encoding a plastid RNA polymerase and modified the steady-state levels of chloroplast gene transcripts. Double mutant analyses indicated that *MDA1* and the previously described *mTERF* genes *SOLDAT10* and *RUG2* act in different pathways. Our findings reveal a new role for mTERF proteins in the response to abiotic stress, probably through perturbed ABA retrograde signalling resulting from a disruption in chloroplast homeostasis.

## Introduction

Chloroplast and mitochondrial genomes are assumed to derive from ancestral prokaryotes that established an endosymbiotic relationship with a primitive eukaryotic cell. Since many genes of those ancestral endosymbionts have been transferred to the nucleus, contemporary plant organelle genomes harbour only 100–200 genes, which encode components of photosynthetic, transcriptional and translational apparatuses. Recent estimations indicate, however, that 3,000 and 2,000 proteins localise in plant chloroplasts and mitochondria, respectively [1]. Most of these proteins are encoded by nuclear genes, synthesised in the cytoplasm and subsequently transported to their target organelle [1]. Therefore, the expression of the nuclear and organellar genomes has to be very precisely coordinated in the plant cell.

The mitochondrial transcription termination factor (mTERF) family was first identified and characterised in humans and other metazoans. Members of this family have been found in monocotyledonous and dicotyledonous plants, and also in the moss *Physcomitrella patens*, but not in fungi and prokaryotes [2]. Metazoan mTERFs are required for the termination and initiation of transcription in mitochondria. Four vertebrate MTERF subfamilies have been described [2], [3], which suggests that mitochondrial transcription regulation is more complex than initially anticipated. Human MTERF1 simultaneously binds to the mitochondrial transcription initiation and termination sites. This creates a DNA loop that promotes the direct delivery of mitochondrial RNA polymerase from the termination to the initiation site, thus accounting for the high mitochondrial rRNA synthesis rate in this organelle [4]. MTERF1 might also modulate mitochondrial DNA replication since the replication pause was sensitive to *MTERF1* over-expression in human cultured cells [5]. Inactivation of the mouse *MTERF2* gene leads to myopathies and memory deficits, which are associated with decreased levels of mitochondrial transcripts and proteins of respiratory chain complexes, thus impairing the respiratory function [6]. In addition, loss of the mouse MTERF3 or the MTERF4 function very early in development is lethal [7], [8]. MTERF3 functions *in vivo* as a repressor of mitochondrial transcription [7], while MTERF4 regulates mitochondrial translation by targeting the methyltransferase NSUN4 to ribosomes [8].

Information on the function of *mTERF* genes in plants is still scarce [9]. An Arabidopsis mTERF protein (PTAC15) has been described as a member of the TAC (*transcriptionally active chromosome*) multi-protein complex involved in the transcription of chloroplast genes [10]. A proteomic study of plastid-enriched nucleoid fractions of maize leaves identified 10 mTERFs among 750 nucleoid-associated proteins [11], indicating a function for mTERFs in the expression of plastid genomes. Three *mTERF* nuclear genes from photosynthetic organisms have been cloned and functionally characterised: *MOC1* (*mterf-like gene of Chlamydomonas1*) in the unicellular alga *Chlamydomonas reinhardtti* [12], and *SOLDAT10* (*SINGLET OXYGEN-LINKED DEATH ACTIVATOR10*; [13] and *BELAYA SMERT* (*BSM*)/*RUGOSA2* (*RUG2*) [14], [15] in Arabidopsis. Perturbation of the MOC1 mitochondrial protein alters the expression profiles of those genes encoding components of mitochondrial respiratory complexes. SOLDAT10 is a chloroplast-localized protein. The *soldat10* mutation alters chloroplast function and suppresses the cell death caused by singlet oxygen in the Arabidopsis *flu* (*fluorescent*) mutant [13]. Of these proteins, only BSM/RUG2 is dually targeted to chloroplasts and mitochondria. The *bsm* and *rug2-1* mutations alter the expression of chloroplast genes at the RNA and protein levels, causing stunted plant growth and paleness [14], [15]. The *bsm* mutant also exhibits arrested embryo development. Besides, impaired *RUG2* function results in decreased levels of mitochondrial transcripts, including those encoding subunits of the respiratory chain [15].

We identified T-DNA alleles of Arabidopsis *mTERF* genes and characterised a member of this family, which we named *MDA1* (for *MTERF DEFECTIVE IN Arabidopsis1*). *MDA1* loss of function alters plant development, leading to defective chloroplast function and gene expression, early flowering, reduced plant growth and pale pigmentation, together with increased tolerance to salt and osmotic stress, and altered responses to sugars and ABA.

## Results

### Identification of *mTERF* Genes in the Arabidopsis and Rice Genomes

To initiate a functional characterisation of the mTERF family of Arabidopsis genes, we performed sequence similarity searches in genome databases and found *mTERF* genes in the Arabidopsis and rice genomes. Rice was chosen to study the conservation of this gene family between dicotyledonous and monocotyledonous plants. We used the amino acid sequence of the Arabidopsis RUG2 protein [15] as a query. In this way, we found 35 and 29 *mTERF* annotated genes in the Arabidopsis and rice genomes, respectively, about as many genes as previously reported by other authors [2], [14]. Most of these genes lack introns: 20 of the 35 Arabidopsis genes and 17 of the 29 rice genes. We confirmed the expression of the *mTERF* genes by searching for their corresponding cDNAs, ESTs [in the *Knowledge-based Oryza Molecular Biological Encyclopedia* (*KOME*; http://cdna01.dna.affrc.go.jp/cDNA/) from rice and *The Arabidopsis Information Resource* (*TAIR*; http://www.arabidopsis.org/)], or MPSS (Massively Parallel Signature Sequencing) data (http://mpss.udel.edu/at/). We found evidence for the transcription of all the genes identified in our searches, except for one Arabidopsis gene (Table S1 and S2). Our analysis of the MPSS data revealed that Arabidopsis *mTERF* genes are expressed in germinating seedlings (16) and in similar numbers in roots (23), vegetative leaves (26), inflorescences (27) and siliques (27). In rice, the highest and lowest numbers of *mTERF* genes were found in young and mature leaves (23) and in mature pollen (4), respectively. Regarding the mTERF proteins, our analysis with SMART (http://smart.embl-heidelberg.de/) showed that they all contain the conserved mTERF motifs, which are characteristic of this family [3]. The average number of mTERF motifs for the Arabidopsis and rice proteins was 6 and 5.4, respectively. TargetP1.1 (http://www.cbs.dtu.dk/services/TargetP/; [16]), PREDOTAR V1.03 (http://urgi.versailles.inra.fr/predotar/predotar.html; [17]), IPSORT (http://hc.ims.u-tokyo.ac.jp/iPSORT/; [18]) and ProteinProwler (http://pprowler.itee.uq.edu.au/pprowler_webapp_1-2/) predicted most of the Arabidopsis and rice mTERFs to be mitochondrial (Table S1 and S2). Thus, TargetP1.1 and ProteinProwler predicted a mitochondrial localisation for 63.3% and 70.0% of the rice and 54.3% and 57.1% of the Arabidopsis mTERF factors, respectively.

### Isolation of Arabidopsis T-DNA Mutants in *mTERF* Genes

We followed a reverse genetics approach in Arabidopsis to identify mutations in the *mTERF* genes with a morphological phenotype. With this purpose in mind, we screened 75 T-DNA insertion lines from different publicly available collections [Table S3; SIGnAL [19]; SAIL (Syngenta Arabidopsis Insertion Library; [20]) and WiscDsLox (https://mywebspace.wisc.edu/groups/Krysan/Web/2010/default.html)] by presumably tagging 29 of the 35 *mTERF* genes of Arabidopsis. Regardless of their phenotype, eight T_3_ plants from each line were transferred to soil and genotyped by PCR for the presence of the annotated T-DNA insertion (Table S4), and the T_4_ plants displaying a mutant phenotype were selected for further studies, thus confirming the mutant phenotype in the T_3_, T_4_ and T_5_ generations.

The N597243 and N819625 lines, putatively carrying a T-DNA insertion in the At4g14605 gene, exhibited a mutant phenotype that was inherited with complete penetrance and constant expressivity. They were backcrossed to the Col-0 wild type and their F_2_ progenies showed a 3∶1 wild-type:mutant segregation ratio (χ^2^ = 0.81, P = 0.37 and χ^2^ = 1.93, P = 0.16, for N597243 and N819625, respectively), indicating that the mutant phenotypes were monogenic and recessive. The presence of insertions in the At4g14605 gene was confirmed by PCR (see Materials and Methods), affecting the fifth exon of the gene (Figure S1A) as annotated on the SIGnAL website (http://signal.salk.edu). For each line, all the F_2_ mutant plants and their F_3_ progenies were homozygous for the T-DNA insertions. The mutant phenotype of the F_1_ plants from an N597243 × N819625 cross further confirmed their allelism. We named these mutants *mda1-1* (*mTERF defective in Arabidopsis1*; N597243) and *mda1-2* (N819625).

### Bioinformatics Analysis of the MDA1 Protein

**Figure 1:**
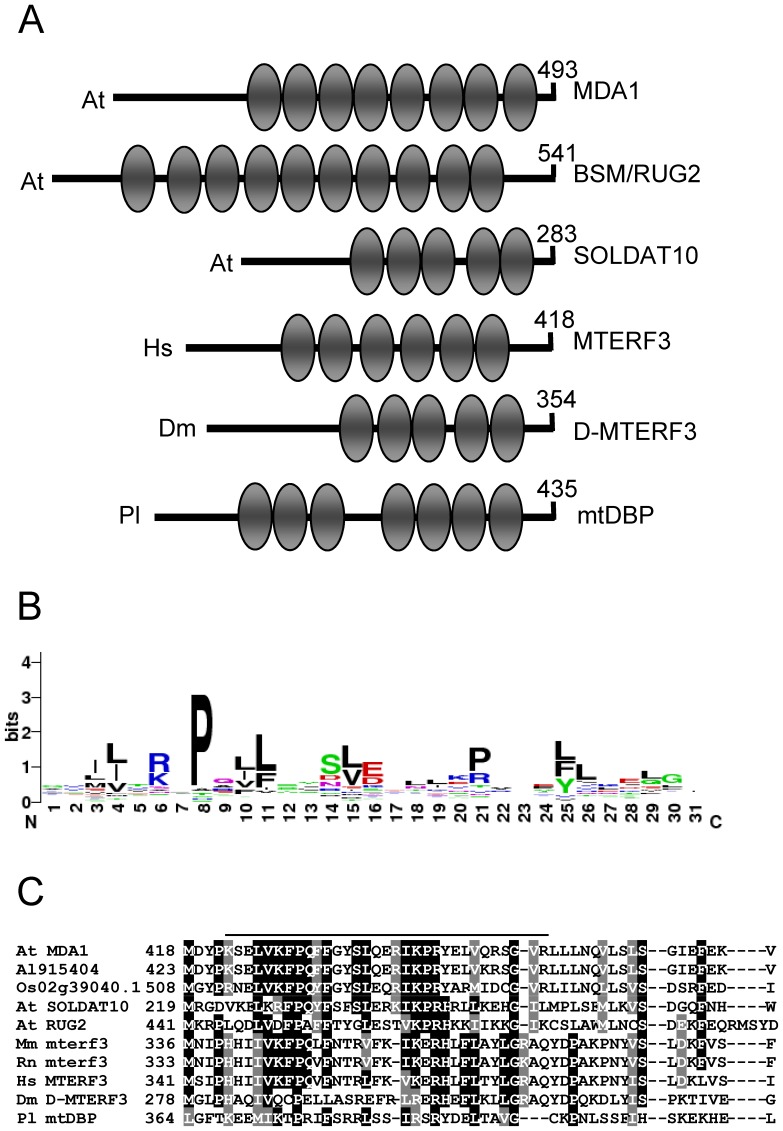
mTERF motifs in members of the mTERF family. (A) Modular architecture of the *Arabidopsis thaliana* (At) mTERF proteins MDA1, BSM/RUG2 and SOLDAT10, human (Hs) MTERF3, *Drosophila melanogaster* (Dm) D-MTERF3 and *Paracentrotus lividus* (Pl) mtDBP. The diagram was drawn using SMART. mTERF motifs are shown as ellipses. The number of amino acids of each protein is indicated. (B) Sequence logo for the mTERF motifs of the above mentioned proteins. Numbers on the abscissa represent positions in mTERF motifs and the ordinates represent the information content measured in bits. The sequence logo was derived using WebLogo (http://weblogo.berkeley.edu/). (C) Multiple alignment of the amino acid sequence of part of the proteins encoded by the At *MDA1*, *SOLDAT10* and *BSM/RUG2*, *Arabidopsis lyrata* Al915404, *Oryza sativa* Os02g39040.1, mouse [*Mus musculus* (Mm)] *Mterf3*, rat [*Rattus norvegicus* (Rn) *Mterf3*], Hs MTERF3, Dm D-MTERF3 and Pl mtDBP genes. Residues conserved across five or more sequences are shaded in black, and similar residues are shaded in grey. Numbers indicate amino acid positions. The alignment was obtained using ClustalX v1.5b. A continuous line indicates an mTERF motif in MDA1.

The *MDA1* gene encodes a predicted protein of 493 amino acids with a molecular mass of 55.9 kDa (http://www.arabidopsis.org/index.jsp). The number of mTERF motifs predicted by SMART was eight (Figure 1A). Database searches allowed us to identify proteins similar to MDA1 in metazoans and plants, but not in archea, eubacteria or fungi. An alignment of the amino acid sequences of the mTERF motifs in MDA1 with those of other plant and metazoan mTERFs (see below) revealed the conservation of a proline residue at position 8 (Figure 1B). The iPSORT, ProteinProwler and TargetP programs yielded a high probability for chloroplast localisation for MDA1 (Table S1), as also reported in the SubCellular Proteomic Database (http://suba.plantenergy.uwa.edu.au/flatfile.php?id). These results are consistent with those obtained by Babiychuk *et al*. [14] using GFP fusions.

The closest identity with MDA1 was found for the ARALYDRAFT_915404 protein from *Arabidopsis lyrata*: 95.6% amino acid identity and 97.8% similarity (Figure 1C). The rice Os02g39040.1 gene product displayed 54.5% amino acid identity and 82.3% amino acid similarity. The amino acid sequence of MDA1 exhibited 31.3% identity and 70.4% similarity to SOLDAT10 [13], and 25.5% identity and 57.8% similarity to BSM/RUG2 [14], [15] (Figure 1C). Other Arabidopsis mTERFs closely related to MDA1 were the products of the At4g38160 (27.8% identity and 65.0% similarity) and At2g44020 (24.6% identity and 59.6% similarity) genes. Furthermore, MDA1 displayed 25.7%, 25.5%, 23.9% and 22.9% overall sequence identity with the human MTERF3, MTERF4, MTERF1 and MTERF2 proteins, respectively, showing a closer similarity to the MTERF3 subfamily members from rat, mouse, sea urchin (*Paracentrotus lividus*) and Drosophila (from 26.6% to 21.7% amino acid identity) than to the remaining subfamily members (Figure 1C). Our results indicate that MDA1 shows closer identities to mTERFs from plants than from metazoans, and hint at a functional conservation of this protein in Arabidopsis and rice.

### *MDA1* Expression Analyses

We examined the expression of the *MDA1* gene in *mda1* mutants. For this purpose, total RNA was extracted from mutant plants on 14 das (days after stratification), reverse-transcribed and PCR-amplified using different primer combinations (Table S4 and Figure S1A). A single band of the expected size was obtained from Col-0 cDNA, but not from *mda1-1* or *mda1-2* cDNAs, by using as primers the oligonucleotides RP and R2a flanking the insertions (Figure S1B). In addition, chimeric transcripts were detected in *mda1* plants when using a primer (F1) which hybridises upstream the T-DNA insertions that disrupt At4g14605 and an LB-specific primer (LBb1.3 or LB1 for *mda1-1* or *mda1-2*, respectively; Figure S1C). The translation of these chimeric transcripts is predicted to yield truncated proteins lacking 110 and 238 C-terminal amino acids in the *mda1-1* and *mda1-2* mutants (deleting 2 and 5 mTERF motifs), respectively. In addition, primers annealing upstream the T-DNA insertions revealed by quantitative RT-PCR (qRT-PCR) down-regulation of *MDA1* expression in *mda1-1* and *mda1-2* compared with Col-0 (2.0-and 1.75-fold, respectively; Figure S2A).

We followed *in silico* and experimental approaches to study the spatial expression of the *MDA1* gene in Arabidopsis. In the first case, we examined the results from different publicly available microarray databases [(Genevestigator (https://www.genevestigator.com/gv/) and the BIO-array resource (BAR; http://bar.utoronto.ca/efp/cgi-bin/efpWeb.cgi)]. We found *MDA1* to be ubiquitously expressed, reaching its highest level of expression in the aerial parts of seedlings, and in young and senescent leaves, and its lowest level in roots and old flowers, according to the tiling array data available at BAR [21], [22]. Similar results were obtained when using Genevestigator. We confirmed the ubiquitous expression of *MDA1* by qRT-PCR. *MDA1* transcripts were detected in all organs analyzed (Figure S2B), with the lowest expression found in roots and the highest in stems and vegetative leaves (2.9-and 2.5-fold higher than in roots, respectively).

### External Morphology and Histology of *mda1* Mutants

**Figure 2:**
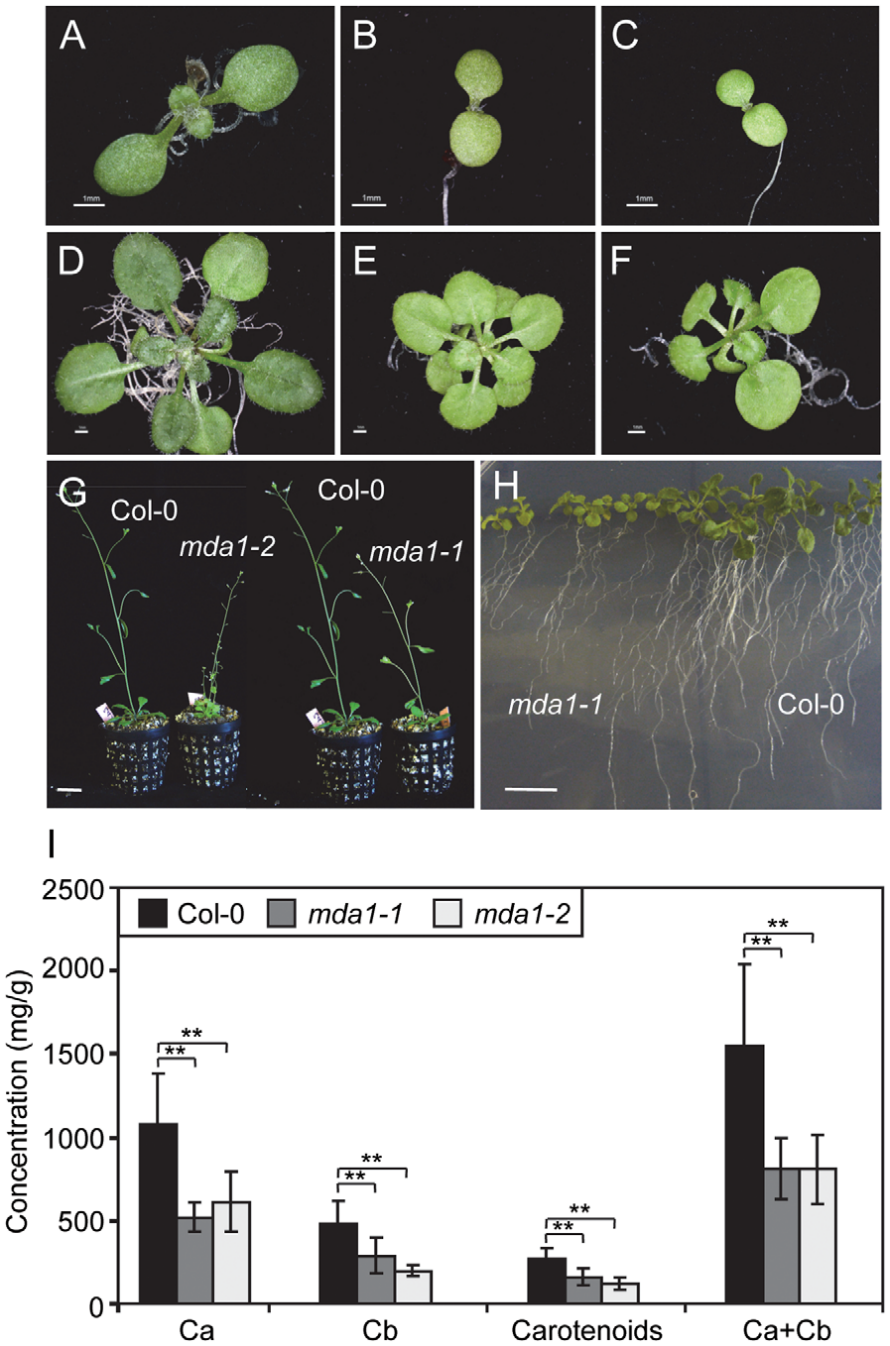
Some morphological traits of the *mda1* mutants. (A–C) Seven-day-old seedlings and (D–F) 21-day-old rosettes of *mda1-1* (B, E), *mda1-2* (C, F) and Col-0 (A, D). (G) Forty-five-day-old plants grown in soil. (H) Fourteen-day-old plants grown on vertically orientated agar plates. Bars  = 1 mm (A–H). (I) Concentration (mg/g of fresh weight) of chlorophyll a (Ca) and b (Cb), and carotenoids in the *mda1* mutants and Col-0. Data represent mean of 10 samples of 15-day-old plants per genotype ± standard deviation (SD). Two asteriscs indicate that the value is significantly different from the wild type at P<0.01, respectively, using Student’s *t*-test.

The most noticeable phenotype of *mda1* plants is the pale pigmentation of their rosette and cauline leaves, stems, sepals and siliques, which was visible early in development. The cotyledons of the *mda1* seedlings exhibited less green pigmentation than the wild type (Figure 2A–C). Consistently, we noted a significant reduction in chlorophyll a, b and carotenoid levels in *mda1* mutants compared with Col-0 (Figure 2I). *mda1* leaves were rounded, their margins lacked indentations and the lamina was uniformly pale (Figure 2D–F). *mda1* plants were small in size if compared with Col-0 plants, as confirmed by measuring several body parameters, which revealed a significant decrease in *mda1* fresh and dry weights, root, hypocotyl and main stem lengths (Figure 2G, H; Table 1). Furthermore, we found that the *mda1* mutations did not affect flower development, although they significantly accelerated flowering, while the *mda1-1* mutation diminished fertility (Table 1).

**Table 1:** Morphometric analysis of the *mda1* mutants.

Body parameters	Genotype		
	Col-0	*mda1-1*	*mda1-2*
Fresh weight^a^	26.1±7.1	9.0±3.0*	9.8±2.9*
Dry weight^b^	2.7±0.8	0.8±0.5*	0.6±0.4*
Root length^c^	75.0±0.3	59.2±6.2*	49.3±4.6*
Hypocotyl length^d,i^	15.3±1.6	13.0±1.2*	12.8±1.3*
Primary stem length^e^	77.0±3.2	36.6±3.8*	27.2±3.9*
Silique length^f^	13.6±0.9	13.1±1.2	N.D.
Number of seeds per silique^g^	50.7±7.3	40.9±7.7*	N.D.
Number of vegetative leaves at bolting^h^	13.4±2.2	12.0±1.1*	9.1±1.1*
Number of days for bolting	27.4±2.5	N.D.	24.2±4.3*

Values shown are the mean of at least 15 measurements ± standard deviation (SD). Lengths are indicated in mm and weights in mg. Measurements were obtained from plant material collected ^a,b^16, ^c^14, ^d^11, ^e^60, ^f,g^42 and ^h^31 days after stratification (das). ^i^Seedlings grown in the dark. *Values were significantly different (Student’s t-test, P<0.01) from those of the wild type (Col-0). N.D.: not determined.

**Figure 3:**
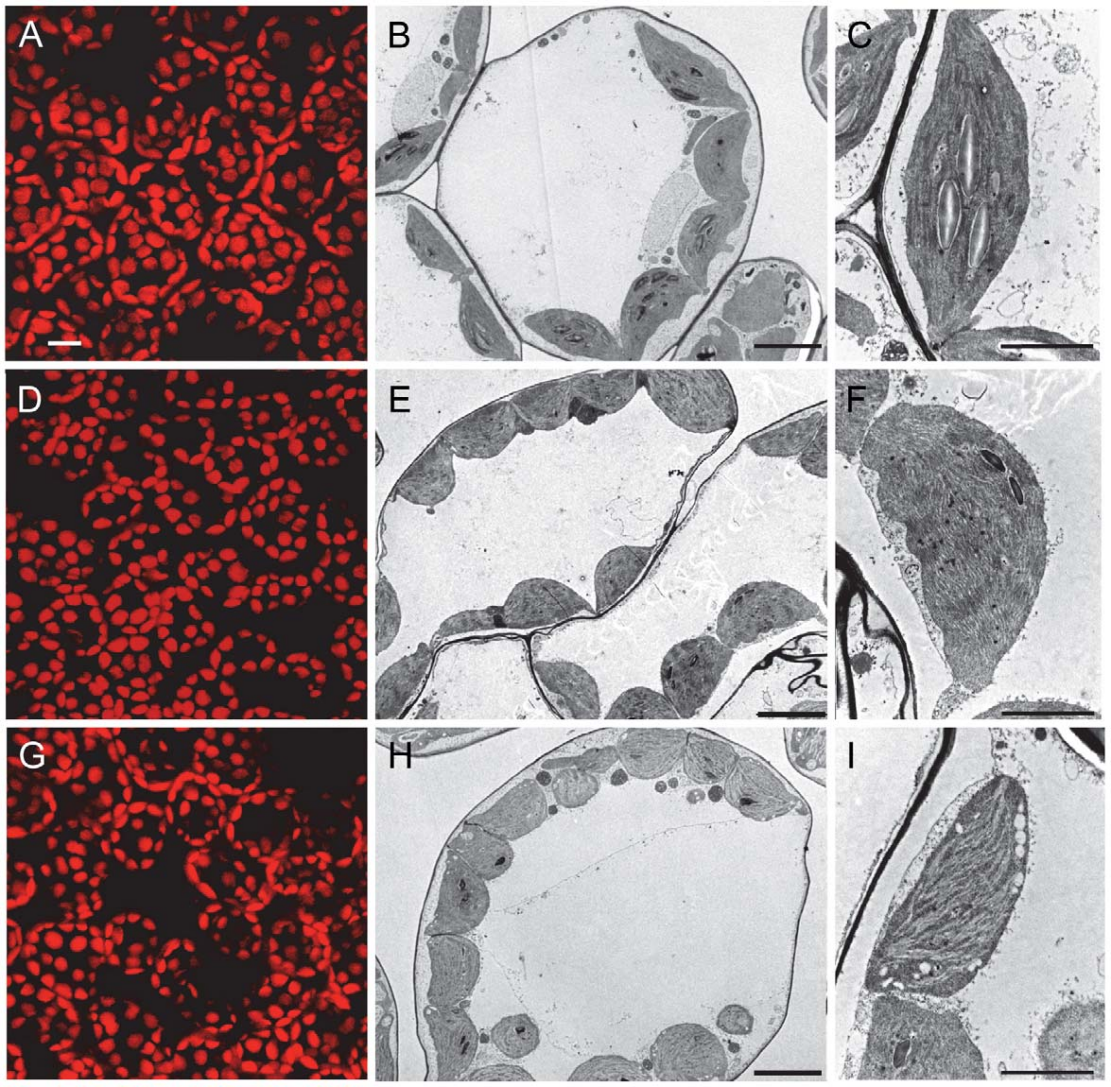
Chloroplast structure in the *mda1* mutants. (A, D, G) Confocal micrographs showing chlorophyll autofluorescence in mesophyll cells of Col-0 (A), *mda1-1* (D) and *mda1-2* (G) third-node leaves. (B, C, E, F, H and I). Transmission electron micrographs of chloroplasts of a Col-0 (B), *mda1-1* (E) and *mda1-2* (H) mesophyll cell. Close-up views of Col-0 (C), *mda1-1* (F) and *mda1-2* (I) chloroplasts. Photographs were taken 21 days after stratification (das). Bars  = 20 µm (A, D, G), 5 µm (B, E, H) and 2 µm (C, F, I).

The internal anatomy of *mda1* leaves was studied by confocal microscopy using chlorophyll autofluorescence of mesophyll cells. We did not find any noticeable differences in the *mda1-1* and *mda1-2* mesophyll cells when we compared them with Col-0, although a slight decrease in chloroplast autofluorescence was observed (Figure 3A, D and G). We examined the leaf chloroplast ultrastructure in both *mda1* and wild-type plants by transmission electron microscopy, which allowed us to identify defects in the *mda1* chloroplast structure (Figure 3B, C, E, F, H and I). Accordingly, chloroplasts in *mda1* mesophyll cells had a low starch grain number, suggesting low photosynthetic activity, as well as enlarged thylakoid lamellas, probably due to a breakdown of thylakoid membranes. Consistently, *mda1* chloroplasts revealed an accumulation of plastoglobuli, storage sites for membrane degradation material (Figure 3C, F and I). Vacuoles were occasionally found in *mda1-2* (Figure 3C and I). Our microscopy studies did not reveal any significant differences in either the number of chloroplasts or the morphology of the mitochondria between *mda1* mutants and Col-0.

### Abiotic Stress Responses of the *mda1* Mutants

Dozens of genes associated with abiotic stress are up-regulated in the Arabidopsis *soldat10* mutant, which displays a very similar morphological phenotype to that of the *mda1* mutants [13]. By employing the BIO-array resource website [21], [22], our expression analysis revealed changes in the *MDA1* transcript levels in those Col-0 plants grown *in vitro* in response to different stress stimuli over time, the most significant being a substantial down-regulation after abscisic acid (ABA) or salt treatments. Besides, we analysed all the Arabidopsis *mTERF* genes using the Arabidopsis whole-genome tiling array express (At-TAX) data (http://www.weigelworld.org/resources/microarray/at-tax) [23] to get a wider perspective on the role of mTERF family members in abiotic stress responses. Most *mTERF* genes (including *RUG2* and *SOLDAT10*) were largely down-regulated in response to ABA, salt or mannitol (Table S5), being these differences usually higher when the exposure of the seedlings to the stress was more prolonged [12 h vs. 1 h; (Table S5)].

**Figure 4:**
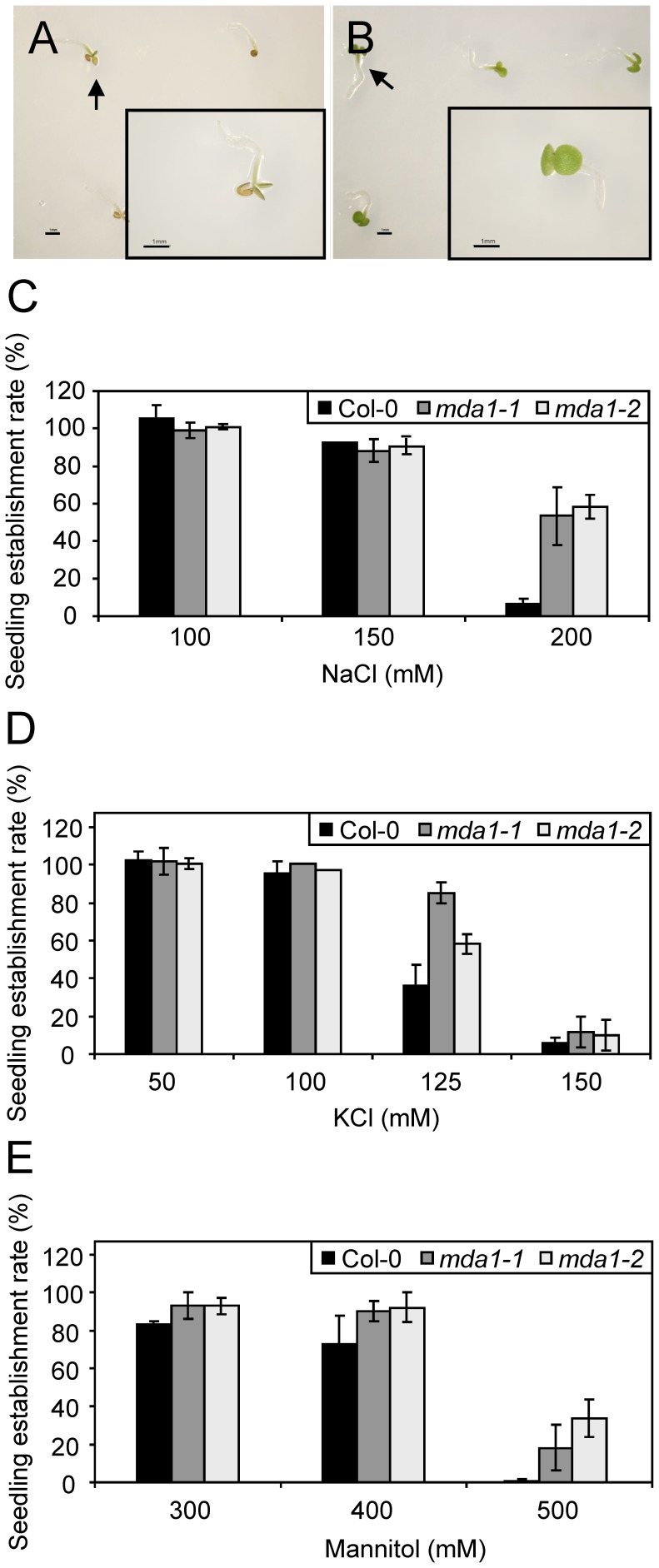
Effects of NaCl, KCl and mannitol on seedling establishment in the *mda1* mutants. (A, B) Col-0 (A) and *mda1-1* (B) seedlings germinating on growth medium supplemented with 200 mM NaCl. The inset images correspond to magnifications of the seedlings indicated by arrows. The mutants display green expanded cotyledons, a phenotypic trait barely observed in Col-0. Scale bars: 1 mm. (C–E) Seedling establishment of the *mda1* mutants and Col-0 in various NaCl (C), KCl (D) and mannitol (E) concentrations. We considered only those seedlings displaying green expanded cotyledons. Error bars represent SD. Each value corresponds to the average of two independent experiments with two to four replicates of 50–100 seeds each. Germination was scored at 10 das.

These results prompted us to study the response of the *mda1* mutants to different agents causing abiotic stress. We first examined the ability of our mutants to form fully expanded green cotyledons at different NaCl concentrations (seedling establishment; Figure 4A, B). We found that *mda1* mutants showed this trait at 200 mM NaCl, a concentration that almost abolished it in Col-0 (53%, 58% and 6% of the *mda1-1*, *mda1-2* and Col-0 seeds, respectively; Figure 4C). Interestingly, we found that 52% of *soldat10* (in a L*er* genetic background) and 13% of L*er* seeds developed expanded cotyledons at 150 mM NaCl, indicating that an impaired *SOLDAT10* function also causes reduced sensitivity to this salt (neither L*er* nor *soldat10* seeds expanded cotyledons at 200 mM NaCl). To determine whether *mda1* mutants were less sensitive to specific ions or osmotic stress than Col-0, we grew them on media supplemented with KCl or mannitol. *mda1* seeds yielded higher rates of seedling establishment than Col-0 on media containing high KCl or mannitol concentrations (Figure 4D, E), indicating that *mda1* mutants are less sensitive than the wild type to K^+^ and Cl^−^ ions and to osmotic stress produced by the osmoticum mannitol.

**Figure 5:**
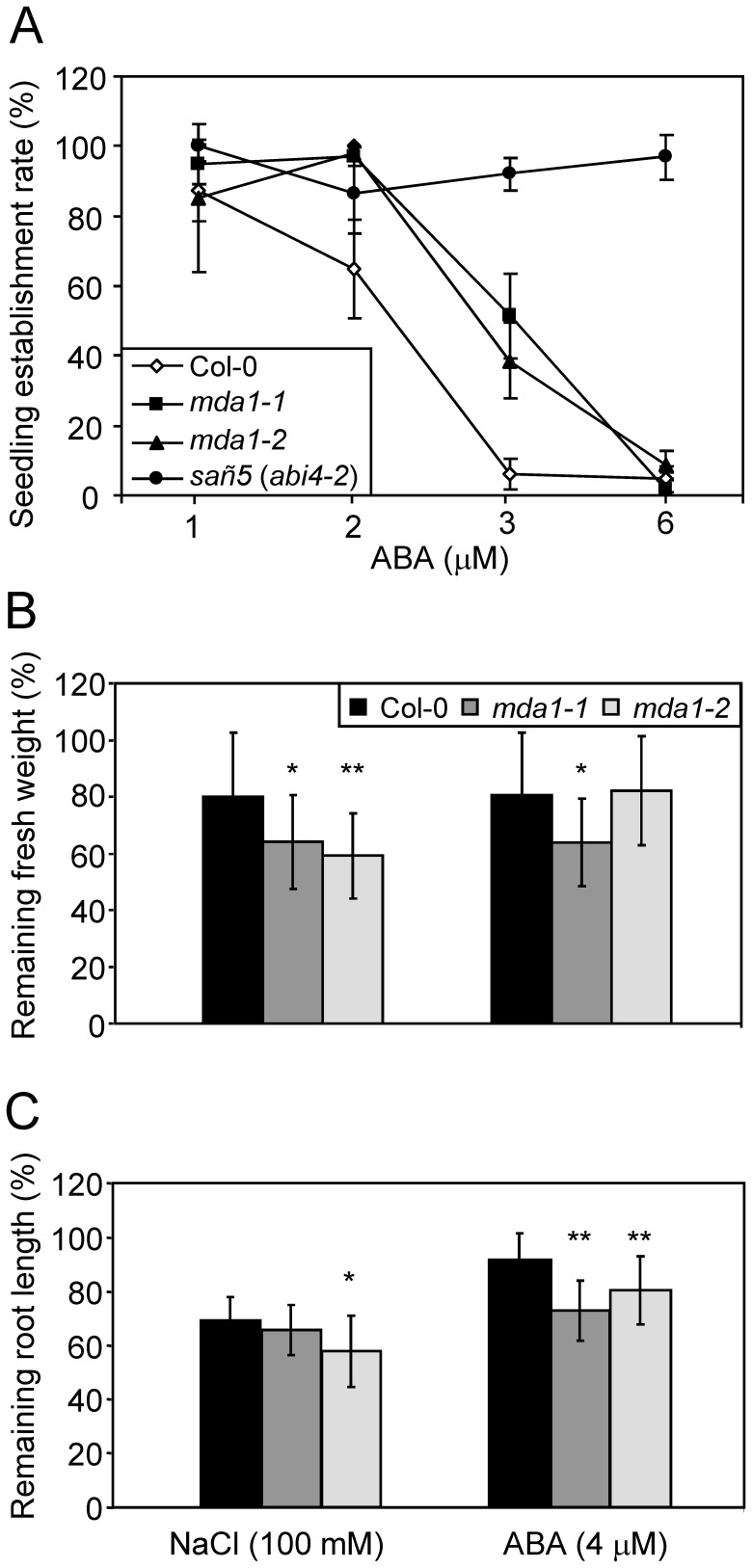
Tolerance to ABA and NaCl of the *mda1* mutants. (A) Effects of different ABA concentrations on seedling establishment in the *mda1* mutants. Data are means of two independent experiments with three replicates of 50–100 seeds each scored 10 das. Error bars represent SD. The *sañ5* (*abi4-2*) ABA insensitive and salt-tolerant mutant was used as a positive control [24]. (B, C) Sensitivity to ABA and NaCl of *mda1* plants. The individuals were transplanted 9 das from non-supplemented growth media to media supplemented with 0, 100 mM NaCl or 4 µM ABA. 12 days after the transfer, tolerance was estimated by determining the fresh weight and root length of the plants transferred to NaCl or ABA supplemented media and referring them to those of the same genotypes transferred to non-supplemented media. These values are represented as percentages of (B) fresh weight and (C) root length of plants transferred to non-supplemented media. Each value corresponds to the mean ± SD of the fresh weight or root length of 15 plants of each genotype. One and two asterisks indicate that the value is significantly different from the wild-type at P<0.05 or P<0.01, respectively, using Student’s *t*-test.

It is well-known that plant responses to environmental stresses are regulated by ABA, a hormone that can inhibit seed germination in response to high ionic and/or osmotic stress produced by salt, cold or drought, and that the mutations affecting ABA synthesis or signalling enhance germination under these stress conditions [24]–[26]. Hence, we investigated the behavior of *mda1* and Col-0 seeds on media containing different ABA concentrations. As depicted in Figure 5A, *mda1* seeds clearly showed higher levels of seedling establishment than Col-0 on medium supplemented with either 2 µM ABA (97%, 98% and 65% of the *mda1-1*, *mda1-2* and Col-0 seeds yielded green expanded cotyledons, respectively) or 3 µM ABA (51%, 38% and 6% of the *mda1-1*, *mda1-2* and Col-0 seeds lead to seedlings with green expanded cotyledons, respectively). Nevertheless, ABA sensitivity was greater in *mda1* than in the null ABA insensitive *sañ5* (also named *abi4-2*) mutant [24] used as a control (Figure 5A). Taken together, these data indicate that the increased tolerance to salt and osmotic stress shown by *mda1* mutants might be explained by their reduced sensitivity to ABA.

To characterise the response of *mda1* mutants to salt and ABA later in development, Col-0 and mutant plants were transferred 9 das from a non-supplemented agar medium to media containing 100 mM NaCl or 4 µM ABA. Their root length and fresh weight were determined after a 12-day growth period and were referred to those of the Col-0 or *mda1* plants transferred at the same time to non-supplemented media. Compared with Col-0, *mda1-1* and *mda1-2* plants subjected to salt stress had a significantly reduced fresh weight (20%, 34% and 41% respectively; Figure 5B) and *mda1-2* also displayed shortened roots (31% and 43% length reduction for Col-0 and *mda1-2*, respectively; Figure 5C). When grown on NaCl, the *soldat10* plants exhibited significantly less fresh weight when compared with L*er* (63.6% and 53.9%, respectively; P = 0.015). On the ABA-supplemented media, *mda1-1* and *mda1-2* exhibited considerably shortened roots compared with Col-0 (28%, 20% and 9%, respectively; Figure 5C), while *mda1-1* individuals displayed significantly lower fresh weight values than Col-0 (63% and 80% of non-stressed plants, respectively; Figure 5B).

We evaluated the effect of temperature stress on *mda1* mutants because we previously reported that the phenotype of the *mTERF*-defective *rug2-1* mutant was temperature-dependent [15]. Growth of *mda1* mutants and Col-0 was similarly affected by culture at 25°C (we normally grow our plants at 20°C; Figure S3D-I). Compared with 20°C, at 15°C (Figure S3A-F) the mutant plants showed reductions in fresh weight significantly higher than Col-0 (65.5%) for *mda1-1* (73.8%), but not significant ones for *mda1-2* (71.9%; Figure S3J). Taken together, our results indicate that *mda1* mutations diminish salt, mild cold and ABA tolerance during vegetative growth.

### Sugar Sensitivity of the *mda1* Mutants

It is known that ABA-deficient or insensitive mutants display altered responses to sugars upon germination (reviewed in [27]). Besides, the Arabidopsis mutant *shs1-1* (*salt hypersensitive1-1*) shows altered sensitivity to NaCl, ABA and sugars, together with inhibition of chlorophyll synthesis, very early in development [28]. Therefore, given the paleness of *mda1* mutants and their reduced sensitivity to salts and ABA during early seedling development, we decided to examine their responses to sugars. Seeds were sown on media containing different concentrations of sucrose (0, 30, 90, 175 and 290 mM) or glucose (330 and 390 mM). We normally grow our mutants on 30 mM sucrose. The *mda1-1* mutant was clearly less sensitive to sugars: on the media containing high concentrations of glucose (330 or 390 mM) or sucrose (290 mM), mutant seedlings developed fully expanded green cotyledons at higher rates than Col-0 (Table 2). Consistently, 18.3% (on 290 mM sucrose) and 51.5% (on 330 mM glucose) of the *mda1-1* germinated seeds, and 5.8% (on 290 mM sucrose) and 4.1% (on 330 mM glucose) of those of Col-0 produced true leaves (observed 14 das). Later in development, paleness of *mda1-1* plants was almost completely suppressed when grown in the presence of 175 mM sucrose (Figure S4A-D). Accordingly, *mda1-1* and Col-0 plants had higher chlorophyll concentrations on 175 than on 30 mM sucrose, and this increase was more marked in *mda1-1* (1.5-fold) than in Col-0 (1.2-fold; Figure S4E).

**Table 2:** Effect of sugars on the germination of the *mda1* mutants.

Genotype	Seedlings displaying green expanded cotyledons (%)						
	Non supplemented	Sucrose (mM)				Glucose (mM)	
		30^a^	90^a^	175^a^	290^b^	330^b^	390^b^
Col-0	98.6±0.4	89.4±3.3	76.7±2.0	83.3±3.4	11.7±2.0	29.8±4.7	2.6±3.2
*mda1-1*	95.4±2.4	93.7±5.5	65.0±0.0	76.9±16.2	31.8±0.7	68.3±12.3	11.2±1.4
*mda1-2*	96.0±0.2	73.0±11.6	71.4±24.0	71.7±16.5	N.D.	31.5±15.8	9.8±4.3

Values shown are the mean ± SD of the percentages of green expanded cotyledons seedlings referred to germinated seeds and obtained in two different experiments with at least 100 seeds per genotype in each experiment. Measurements were performed ^a^4 and ^b^10 das. N.D.: not determined.

### Effects of *mda1-1* Mutation on the Expression of Plastid and Nuclear Genes

Considering that MDA1 is an mTERF-plastid protein which is putatively involved in transcriptional control and that the mutations in the previously characterized *mTERF* genes *SOLDAT10* and *BSM/RUG2* modify the levels of chloroplast transcripts, we decided to study the expression of several plastid genes by qRT-PCR using the RNA extracted from seedlings 14 das. We selected genes whose expression is known to be affected in the *soldat10, rug2-1* and/or *bsm* mutants. Besides, we included representatives of the three classes of genes that are transcribed by different RNA polymerases: only the plastid-encoded polymerase (PEP; class I), PEP and the nuclear-encoded polymerase (NEP; class II), and only NEP (class III) [29]. According to these criteria, the expression of the *psbA* [(class I) encoding a core subunit of the photosystem II], *matk* [(class II) encoding a maturase], *clpP* [(class II) encoding the proteolitic subunit of the Clp ATP-dependent protease], *rpoB, rps18* and *accD* [(class III) encoding the core β subunit of PEP, a ribosomal protein and a subunit of the acetyl-Coa carboxylase for lipid biosynthesis, respectively] genes was studied. In comparison to Col-0, in *mda1-1,* we found significant differences in the expression of *psbA*, *accD*, *rps18* [2.2-fold (2.2±0.6; P = 0.01), 1.7-fold (1.7±0.6; P = 0.04) and 1.6-fold (1.6±0.3; P = 0.01) up-regulated, respectively] and of *clpP* [1.7-fold down-regulated (0.6±0.2; P = 0.008)], whereas the transcript levels of *matk* [1.3-fold (1.3±0.5; P = 0.2)] and *rpoB* (0.9±0.1; P = 0.3) were only slightly affected.

Defective chloroplast development may modify the expression of nuclear genes through retrograde signalling [30]. Consequently, the transcript levels of the *RpoTp/SCA3* nuclear gene, encoding a plastid RNA polymerase [31], change in Arabidopsis mutants which are impaired in plastid development, such as *rug2-1* [15]. For this reason, we investigated whether the expression of *RpoTp/SCA3* was affected in *mda1* mutants: *RpoTp/SCA3* was found significantly up-regulated in *mda1-1* (1.7±0.1; P = 0.01) and *mda1-2* (1.6±0.3; P = 0.008) when compared with Col-0.

### Genetic Interactions among *mTERF*-defective Mutants

To identify the genetic interactions among the *mTERF* genes whose perturbation led to a mutant phenotype, we crossed *mda1* mutants with *rug2-2* [15] and *soldat10* [13] in the Col-0 and L*er* genetic backgrounds, respectively. In all cases, we confirmed the phenotypes of the double mutants identified in F_2_ by studying the F_3_ progenies derived from selfed F_2_ plants displaying single or double mutant phenotypes, whose genotypes were verified by PCR (see Materials and Methods).

**Figure 6:**
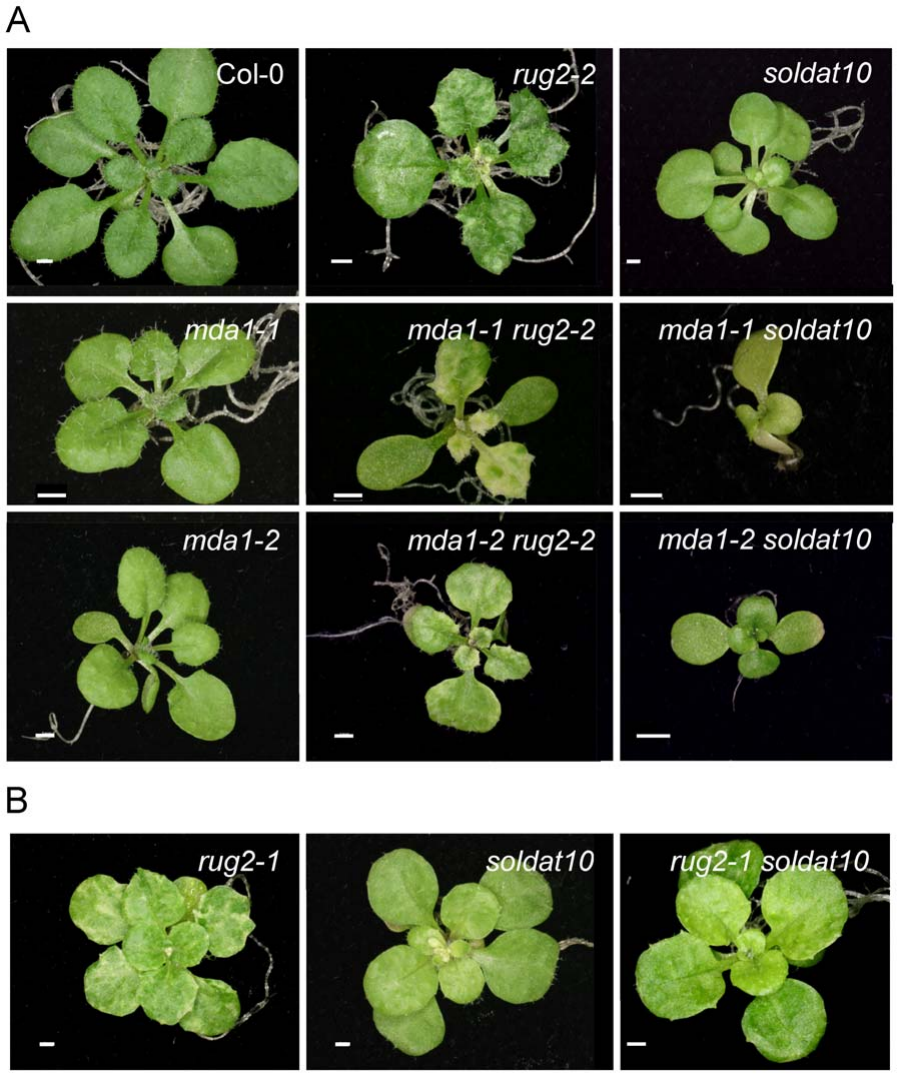
Genetic interactions between *mTERF* mutants. (A) Additive phenotypes of *mda1 rug2-2* and *mda1 soldat10* double mutants. Rosettes from a wild-type (Col-0) and the single mutants *rug2-2*, *mda1-1* and *mda1-2* (in a Col-0 genetic background), *soldat10* (in a L*er* genetic background) and the *mda1-1 rug2-2*, *mda1-1 soldat10*, *mda1-2 rug2-2* and *mda1-2 soldat10* double mutants. (B) Genetic interaction between *rug2-1* and *soldat10*. Rosettes from the *rug2-1* and *soldat10* single mutants, both in a L*er* genetic background, and the *rug2-1 soldat10* double mutant. Pictures were taken 21 das. Scale bars: 1 mm.

The *rug2-2* × *mda1-1* and *rug2-2* × *mda1-2* crosses allowed us to identify additive double mutant phenotypes in their F_2_ progenies since they exhibited a combination of phenotypic traits from their corresponding parentals (Figure 6A). Since *MDA1* and *RUG2* are linked, the four phenotypic classes found did not fit the expected 9∶3:3∶1 ratio. As regards the *mda1-1* × *soldat10* and *mda1-2* × *soldat10* crosses, we identified double mutants with a clearly additive phenotype in all the F_2_ progenies studied: they had much smaller leaves and rosettes than *mda1* or *soldat10* (Figure 6A). Only three phenotypic classes were found in the F_2_ progenies (wild-type, single or double mutant), probably because of the similarity between the phenotypes of *soldat10* and *mda1*. Additivity of the phenotypes caused by *soldat10* and *mda1* was confirmed by analysing the F_3_ progenies. We also crossed *rug2-1* with *soldat10*, both in the L*er* genetic background, and the F_2_ progeny showed a 9∶3:3∶1 segregation ratio (χ^2^ = 0.97; *P* = 0.82), and the F_2_ and F_3_ double mutants were smaller and paler than their single mutant siblings (Figure 6B), a phenotype which we interpreted as additive.

We evaluated the response of the double mutants to ABA by measuring root growth and seedling establishment. Root growth assays revealed that the *rug2-1* and *soldat10* mutations increase ABA sensitivity whereas no differences were found for *rug2-2* compared with Col-0 (Table 3). On ABA, root length of the *mda1-2 rug2-2* plants was similar to that of *mda1-2* whereas it was shortened in *rug2-1 soldat10* and *mda1-1 soldat10* over that of the single mutants, which suggests additivity (Table 3). Regarding seedling establishment on ABA, *rug2-1*, *soldat10* and the *rug2-1 soldat10* double mutant were as sensitive as L*er*. On the contrary, the *rug2-2*, *mda1-2 rug2-2* and *mda1-2 soldat10* seedlings were more tolerant than the corresponding wild type (Table 3). The rate of seedling establishment on ABA for *mda1-2 rug2-2* was similar to that of the single mutants, whereas *mda1-2 soldat10* exhibited higher and lower rates than *soldat10* and *mda1-2*, respectively, likely due to its mixed genetic backgrounds (Table 3).

**Table 3:** Tolerance to ABA of the double mutants.

Genotype	Remaining root length (%)^a^(4 µM ABA)	Seedling establishment (%)^b^(3 µM ABA)
Col-0	91.4±9.8	6.3±4.4
*mda1-1* ^c^	72.7±11.2**	53.4±9.5
*mda1-2* ^c^	80.2±12.5**	38.4±10.9
*rug2-2* ^c^	93.9±15.7	39.6±10.9
*mda1-2 rug2-2* ^c^	80.7±17.3*	32.8±5.2
L*er*	77.3±19.0	1.9±2.7
*rug2-1* d	58.8±17.1*	0
*soldat10* d	67.0±14.6	1.1±1.3
*rug2-1 soldat10* d	49.3±10.1**	0
*mda1-1 soldat10* e	53.3±23.4**^f^	N.D.
*mda1-2 soldat10* e	69.5±24.7**^g^	21.1±12.5

a These values are represented as percentages of root length of plants transferred to non-supplemented media as described in Methods. Each value corresponds to the mean ± SD of the root length of 15 plants of each genotype.

b Values shown are the mean ± SD of the percentages of green expanded cotyledons seedlings of two replicates of 50 seeds each scored 10 das. N.D.: not determined.

c–e Mutant genotypes in a ^c^Col-0,

d L*er* or

e L*er*/Col-0 mixed genetic background, respectively.

f,g Differences were significant from

f Col-0 and L*er* or ^g^only from Col-0, respectively. Values were significantly different from the corresponding wild type at *P<0.05 or **P<0.01 using Student’s *t*-test.

We studied by qRT-PCR the expression of the nuclear *RpoTp/SCA3* and plastid *psbA* genes, in the *mda1-1 rug2-2* and *mda1-1 soldat10* double mutants. In *mda1-1 rug2-2* compared with Col-0, *RpoTp/SCA3* (2.3±0.2; P = 0.008) and *psbA* (2.8±0.6; P = 0.05) were up-regulated to levels slightly higher than those of *mda1-1* (see above). *psbA* was up-regulated in *mda1-1 soldat10* compared with Col-0 (2.5±0.8; P = 0.008) or L*er* (2.5±0.8; P = 0.01) at a similar extent than *mda1-1* (see above). *RpoTp/SCA3* expression was clearly dependent on the genetic background; it was up-regulated in Col-0 (2.8±0.2; P = 0.006) compared with L*er.* This would explain the differences found in *RpoTp/SCA3* expression between *mda1-1 soldat10* and either L*er* (1.9-fold up-regulated) or Col-0 (1.3-fold down-regulated).

## Discussion

Although chloroplasts and mitochondria are essential for life, and despite experimental and *in silico* studies estimating the number of proteins which are located in these organelles to run into thousands, knowledge of their role in plant biology is limited. To help elucidate the function of the nuclear-encoded proteins localised to chloroplasts and/or mitochondria, we studied the Arabidopsis mTERF family of transcriptional regulators. To this end, we first searched for members of this gene family in the genomes of the dicotyledonous *Arabidopsis thaliana* and the monocotyledonous *Oryza sativa*. Our bioinformatics analyses revealed that the numbers of annotated *mTERF* genes in the Arabidopsis and rice genomes are similar and substantially higher than in metazoan genomes (four in vertebrates, three in *Drosophila melanogaster* or one in *Caenorhabditis elegans* [2], [14]). We found experimental evidence for the expression of these genes, thus validating the *in silico* identification. Furthermore, our results suggest that Arabidopsis and rice mTERFs are targeted to chloroplasts or mitochondria, most of which are potentially located in mitochondria. This has been experimentally confirmed in Arabidopsis by GFP fusions [14]. In animals, mTERF proteins are mitochondrial, and their molecular characterisation has revealed their participation in mitochondrial transcription initiation, termination, translation and mtDNA replication. We speculate that the large number of *mTERF* genes in plants might be explained by them requiring the accurate expression of not only mitochondrial, but also plastid genes. Besides, mTERFs might differentially contribute to regulate the organelle gene expression in distinct developmental stages and tissues, or in response to environmental demands or stress.

Our reverse genetics approach underpins the importance of the mTERF gene family in Arabidopsis, as previously shown throughout the characterisation of the *mTERF*-related genes *SOLDAT10* [13] and *BSM/RUG2* [14], [15]. *mda1* insertional alleles curtail *MDA1* expression. Besides, they would encode truncated proteins lacking 110 (*mda1-1*) or 238 (*mda1-2*) residues from the C-terminus, which would likely include divergent amino acids translated from T-DNA because we detected chimeric transcripts. This suggests that *mda1* mutants are not null. Despite all this, the *mda1-1* and *mda1-2* phenotypes are indistinguishable, which might be explained by the existence of a redundant function supplying *MDA1* deficiency regardless of the extent of mutational damage. Our morphological, physiological and molecular analyses of the *mda1 soldat10* and *mda1 rug2-2* double mutants suggest additivity rather than synergy which would rule out *RUG2* or *SOLDA10* as that redundant function. This indicates that the *mTERF* genes so far characterised in Arabidopsis participate in different pathways. Alternatively, the mTERF domains remaining in *mda1-2* may be sufficient to accomplish a level of activity similar to that of *mda1-1*.

Consistent with the chloroplast targeting of the MDA1 protein, loss-of-function *mda1* alleles alter chloroplast morphology and cause disorganised thylakoid membranes and a reduction in starch grains, indicating diminished photosynthetic activity. Impaired chloroplast activity results in reduced chlorophyll levels in *mda1* plants, leading to pale green organs and general stunted growth, as shown by the lower weight and reduced height of *mda1* plants. In line with the pleiotropic effects of *mda1*, the *MDA1* gene was broadly expressed. Leaf morphology was almost normal in *mda1* mutants, except for their roundness. Stunted plant growth, chlorophyll levels and green pigmentation are phenotypic traits shared by other previously characterised *mTERF* mutants, such as *rug2* [15] and *soldat10* [13]. Like *mda1*, *rug2-1* also showed abnormal chloroplasts. Interestingly, the short hypocotyls of *mda1* plants suggest that *MDA1* is required to complete the etiolated developmental program in the dark and also suggests a role for this gene in etioplasts. The most severe phenotype described so far for an *mTERF*-defective mutant was displayed by *bsm*, which is likely a null allele of the *BSM/RUG2* gene, exhibiting albino cells and severe alterations in organogenesis. In addition, *bsm* cells require hormone supplementation to proliferate as well as to grow *in vitro* [14]. Like *bsm*, *rug2-1* and *soldat10*, *mda1-1* plants exhibit an altered plastid gene expression. The fact that the *mda1-1* mutation modifies the transcript levels of those genes transcribed by NEP, PEP or both, suggests that MDA1 might be directly or indirectly required by different plastid transcriptional machineries for appropriate gene expression.

The proper activity of the different plant cell genomes entails tight coordination. Thus, retrograde pathways transmit the developmental, metabolic or physiological chloroplast status to the nucleus by modifying the expression of those nuclear genes whose products act on chloroplasts, such as the *RpoTp/SCA3* gene, which encodes a plastid RNA polymerase [31]. Accordingly, we found that *RpoTp/SCA3* up-regulates in *mda1* plants as in Arabidopsis *rug2-1* [15] and *rpoT;2* (affected in the chloroplast and mitochondria-targeted RNA polymerase; [32]) mutants and the plastid-ribosome deficient *albostrians* mutant from barley [33]. This up-regulation may attempt to compensate for the defective plastid function caused by the altered plastome expression in mutants.

Our findings reveal that disrupted MDA1 activity causes altered responses to abiotic stresses, according to the *in silico* microarray-based results, and they support previous observations suggesting a role for mTERFs in plant stress. Thus, *soldat10* is constitutively adapted to light stress [13], whereas a *Brassica napus mTERF* gene and its Arabidopsis orthologue are up-regulated under abiotic stress conditions [34]. Consistent with this, our analysis using the At-TAX tiling array data showed differences in the expression of most Arabidopsis *mTERF* genes after salt, mannitol or ABA treatments, suggesting a role for mTERFs in abiotic stress responses. *mda1* mutants exhibit reduced seed sensitivity to the inhibition of seedling establishment caused by high NaCl, KCl or mannitol concentrations, yielding higher rates than those of Col-0 under stress conditions. As ABA plays a central role in plant adaptive responses to environmental stresses, and since the perturbation of ABA signalling in Arabidopsis *abi* (*ABA insensitive*) mutants results in increased salt tolerance [24], [35], we evaluated the *mda1* response to ABA. We found *mda1* early seedling development to be less sensitive to this hormone, indicating that the *MDA1* function is required for a proper ABA response in this stage. The experimental results support that there is an interaction between sugar and ABA signalling during early seedling development in Arabidopsis, to the extent that ABA biosynthesis and signalling pathways are at least partially modulated by glucose (for reviews, see [36]–[38]). Consequently, the mutants identified in screens for an altered sugar response were actually allelic to *aba2* (*ABA deficient2*) or *abi4* mutants [27]. Therefore, we decided to investigate if *mda1* mutants, which are partially insensitive to ABA, also show an altered sugar response. We found this to be the case because, in the presence of high glucose or sucrose concentrations, *mda1-1* seeds yielded higher seedling establishment rates than Col-0.

Altered sugar, salt and ABA responses during early seedling development were also observed in the Arabidopsis *shs1-1* mutant [28]. SHS1 belongs to the mitochondrial carrier family of proteins involved in the energy transfer being located in plants in plastids [39] or the endoplasmic reticulum [28]. We considered reduced sensitivity to ABA to be a likely explanation for the altered responses to salts, sugar and osmotic stress exhibited by *mda1* mutants because, as previously mentioned, any perturbation in ABA-mediated perception mechanisms might alter stress responses. Consistently with the phenotypes that we found, a recent study has reported that the *mTERF* genes play an important role in germination: an in-depth transcriptomic profiling at 10 time points during Arabidopsis germination identified a total of 15,789 genes to be expressed, including 23 *mTERFs* [40]. Moreover, 15 of the 23 *mTERF* genes identified, including *MDA1*, were present in a subgroup of 775 genes, which were transiently expressed during germination [40]. Furthermore, we found that *mda1* mutations enhanced NaCl, mild cold, ABA or sugar sensitivity during vegetative growth, and that they extended MDA1 requirement for adaptation to abiotic stresses later in development.

Apart from germination and seedling establishment, flowering time, the other fundamental developmental transition, is also influenced by *mTERF* genes. Accordingly, *mda1*, *soldat10* (data not shown) and *rug2* [15] mutants are early flowering, which is in agreement with the finding that germination and flowering share genetic controls [41]. mTERFs might affect flowering by their participation in ABA plastid signalling (see below) given that a role for ABA as a floral repressor has been proposed [42], although contradictory results have been reported [43].

Linking *mda1* altered response to ABA with the function of the perturbed gene as a putative modulator of gene expression in chloroplasts is no straightforward task. Nevertheless, we speculate that as the initial ABA biosynthesis steps take place in chloroplasts, a branch of plastid-specific ABA perception and/or signal transduction might be localised within this organelle and can, hence, connect salt, cold or water stress perception to the ABA response. In fact, a role as a putative ABA receptor has been attributed to the Arabidopsis Mg-chelatase H subunit (ChlH), a chloroplast protein involved in chlorophyll biosynthesis which is also known as ABAR (ABA receptor; [44], [45] or GUN5 (GENOMES UNCOUPLED 5), a regulator of plastid-to-nucleus retrograde signalling [46]. Furthermore, it has been proposed that chloroplasts would be particularly placed to act as environmental sensors in perceiving stress and in coordinating the expression of those nuclear genes encoding adaptive stress response through retrograde signalling [47]. Therefore, we hypothesise that impaired *mTERF* activity would disrupt chloroplast homeostasis and negatively affect ABA retrograde signalling which elicits the nuclear-encoded functions required to cope with environmental stress. A role in plastid signalling has been associated with ABA: the ABI4 transcription factor participates in plastid and mitochondria retrograde signalling downstream of these pathways [48], [49]. Moreover, low ABA concentrations positively regulate plastid differentiation by promoting plastid gene expression in etiolated or light-grown seedlings [50]. Interestingly, the albino cells of the *bsm* mutant accumulate THIOGLUCOSIDE GLUCOHYDROLASE1 [14], also known as myrosinase, a protein required for ABA inhibition of stomatal opening [51]. This reinforces the connection we found between mTERFs and ABA responses during stress.

In conclusion, our analysis of a new mTERF factor shows the importance of this gene family in plants and uncovers its role in abiotic stress responses, probably through ABA signalling, in connecting chloroplast gene expression, ABA activity and plant adaptation to stress.

## Materials and Methods

### Plant Material and Growth Conditions

Cultures and crosses were performed as described by Ponce *et al.* [52] and Berná *et al.* [53], respectively. The seeds of the *Arabidopsis thaliana* (L.) Heynh. wild-type accessions Landsberg *erecta* (L*er*) and Columbia-0 (Col-0) were obtained from the Nottingham *Arabidopsis* Stock Centre (NASC). The seeds of the T-DNA insertion lines (Table S3) were provided by the NASC and are described on the SIGnAL website ([19]; http://signal.salk.edu). The *rug2-1* mutant was isolated in a L*er* background after ethyl methanesulphonate (EMS) mutagenesis [53] and was backcrossed twice to the wild-type L*er* [15]. The *rug2-2* mutant in a Col-0 genetic background was characterised in a previous work [15]. The *soldat10* seeds in a L*er* genetic background were kindly provided by Klaus Apel (the Boyce Thompson Institute for Plant Research, Ithaca, NY). Pigment extraction and quantification were carried out as previously described [31].

### Bioinformatics Analyses

Amino acid sequence comparisons and similarity searches were performed by using FASTA (http://fasta.bioch.virginia.edu/fasta_www2/fasta_www.cgi; [54]) and BLAST (http://blast.ncbi.nlm.nih.gov/Blast.cgi; [55]).

### Morphological and Ultrastructural Analyses

Dry weight was measured in plants that were oven-dried overnight at 55°C. Root, silique, stem and hypocotyl measurements were obtained with the ImageJ program (http://rsb.info.nih.gov/ij/docs/menus/file.html) from pictures taken by a Leica MZ6 stereomicroscope equipped with a Nikon DXM1200 digital camera. A Student’s *t*-test was applied to the data obtained, with a significance level of 0.01. Confocal imaging was performed as described by Hricová *et al.* [31]. For transmission electron microscopy, mutant and wild-type plant material was harvested at the same time of the day and prepared as described by Hricová *et al.* [31]. Samples were visualised under a Zeiss EM10C transmission electron microscope.

### Identification of the T-DNA Insertions in Mutant Lines

To genotype mutants, we extracted DNA from the T_3_, T_4_ and T_5_ mutant plants, from the F_2_ segregating plants derived from the backcrosses, and from the F_1_ plants derived from the complementation analyses. The DNA was PCR-amplified using the primers (RP and LP) designed by the *T-DNA Primer Design* (http://signal.salk.edu/tdnaprimers.2.html) tool, which were hybridised with the genomic sequences flanking the insertions in combination with the T-DNA specific primers LB1 or LBb1.3 (Table S4).

### RNA Extraction and Semi-quantitative RT-PCR

Total RNA was extracted from ≈80 mg of the Col-0, *mda1-1* and *mda1-2* 14 das seedlings using TRIsure (Bioline) and treated with DNase I following the manufacturer’s instructions. RNA was ethanol-precipitated and resuspended in 40 µl of RNase-free water. Two to four micrograms of each sample were reverse-transcribed using random hexamers, and PCR amplification of first strand cDNA was performed as described by Quesada *et al.* [56]. Next, 1 µl of the resulting cDNA solution was used for the qRT-PCR amplifications. To detect the *MDA1* transcripts in the Col-0 and *mda1* mutants, different primers combinations were used as described in Table S4 and Figure S1A.

### Quantitative RT-PCR

Total RNA was extracted from 50–70 mg of 14-day-old Col-0, L*er*, *mda1-1*, *mda1-2*, *mda1-1 rug2-2* and *mda1-1 soldat10* seedlings, 3-week-old roots and vegetative leaves, and 45-day-old stems, cauline leaves and flowers and it was treated with DNase I following the manufacturer’s instructions. RNA was ethanol-precipitated and resuspended in 40 µl of RNase-free water. Two micrograms of RNA from each sample were reverse-transcribed using random hexamers, as described by Quesada *et al.* [56]. cDNAs were diluted three times with water and 1 µl of the resulting solution was used for qRT-PCR amplifications, which were carried out in an ABI PRISM 7000 Sequence Detection System (Applied Biosystems). Oligonucleotides (Table S4) were designed as described in Quesada *et al*. [15]. Each 25-µl reaction mix contained 12.5 µl of the SYBR-Green/ROX qPCR Master Kit (Fermentas), 0.4 µM of primers and 1 µl of the cDNA solution. Relative quantification of the gene expression data was performed by following the 2^−ΔΔC^_T_ method [57]. Each reaction was done in two or three replicates and three different biological replicates were used. The expression levels were normalised to the C_T_ values obtained for the housekeeping *OTC* gene [56].

### Seedling Establishment and Growth Sensitivity Assays

For the seedling establishment assays, sowings were conducted by plating seeds in a water suspension using a Pasteur pipette at a density of 100 regularly spaced seeds per Petri dish of GM agar medium (Murashige and Skoog medium containing 1% sucrose) supplemented with NaCl (0–200 mM), KCl (0–150 mM), mannitol (0–500 mM), ABA (1.5–6 µM), sucrose (30–290 mM) or glucose (330 and 390 mM). Seed germination was scored at 4, 10 or 14 das on Petri dishes kept at 20±1°C under 72 µmol m^−2^ s^−1^ of continuous light, considering that only those seedlings exhibiting green and fully expanded cotyledons displayed seedling establishment.

To evaluate the salt and ABA responses during later stages of plant growth, seeds were sown on Petri dishes containing GM agar medium. The seedlings were transferred 9 das to new plates containing agar medium supplemented with 100 mM NaCl or 4 µM ABA. Tolerance was estimated by determining plant fresh weight and root length after 12 days of stress treatment and by referring the values to those of plants transferred to non-supplemented media. Temperature-sensitivity assays were performed as previously described [31].

## Supporting Information

Figure S1 **Detection of** ***MDA1*** **transcripts in Col-0 and** ***mda1*** **mutants.** (A) Structure of the At4g14605 (*MDA1*) gene indicating the positions of the T-DNA insertions in *mda1-1* and *mda1-2* by triangles. Boxes and lines indicate exons and introns, respectively. White boxes correspond to the 5′ and 3′ untranslated regions. Oligonucleotides used to study *MDA1* expression are represented by horizontal arrows (not drawn to scale; Table S4). (B–C) PCR amplifications were performed using genomic DNA (gDNA) or complementary DNA (cDNA) from 2-week-old plants and primers hybridizing with (B) genomic sequences flanking the insertions in the *mda1* mutants or (C) the LB of the T-DNAs and the upstream genomic region. The *OTC* gene was used as an internal control [56].
(PPT)

Figure S2 **Quantitative RT-PCR analysis of the expression of the** ***MDA1*** **gene.** (A) Level of expression of the *MDA1* gene in the wild type (Col-0) and the *mda1* mutants and (B) in different organs of Col-0 plants after normalization with those of the *OTC* gene (see Material and methods). Bars indicate relative levels of expression, determined as 2^−ΔΔCT^. A value of 1 is assigned to *MDA1* expression in Col-0 (A) and roots (B). Error bars indicate the range of variation of the 2^−ΔΔCT^ values, obtained using three different biological replicates and triplicate reactions.
(PPT)

Figure S3 **Effect of temperature on the growth of the** ***mda1*** **mutants.** Representative plants are shown for (A, D, G) Col-0, (B, E, H) *mda1-1* and (C, F, I) *mda1-2*, which were grown at 15 (A–C), 20 (D–F) or 25°C (G–I). (J) Fresh weight of Col-0 and *mda1* individuals grown 21 days at 15°C. Each value represents the percentage of fresh weight of unstressed plants (grown at 20°C). Error bars indicate the mean ± standard deviation (SD) of the fresh weight of 15 plants of each genotype. An asterisk indicates that the value is significantly different from the wild type at P<0.05 using Student’s *t*-test. Pictures were taken 21 das. Scale bars indicate 1 mm.
(PPT)

Figure S4 **Sugar sensitivity of the** ***mda1-1*** **mutant.** Pictures are shown for (A, B) Col-0 and (C, D) *mda1-1* plants grown (A, C) in the presence of 30 mM or (B, D) 175 mM of sucrose. (E) Concentration (mg/g of fresh weight) of chlorophyll a (Ca) and b (Cb) in Col-0 and *mda1-1* plants grown on media supplemented with 30 or 175 mM of sucrose. Data represent mean of 10 samples of 15-day-old plants per genotype ± SD. One and two asterisks indicate that the values are significantly different at P<0.05 or P<0.01, respectively, using Student’s *t*-test. Pictures were taken 21 das. Scale bars indicate 1 mm.
(PPT)

Table S1 **Arabidopsis mTERF proteins.**
(DOC)

Table S2 **Rice mTERF proteins.**
(DOC)

Table S3 **T-DNA lines for** ***mTERF*** **genes used in this work.**
(DOC)

Table S4 **Primers used in this work.**
(DOC)

Table S5 **Tiling array expression for Arabidopsis** ***mTERF*** **genes.**
(DOC)
