# Supplementary material for: Arabidopsis MDA1, a Nuclear-Encoded Protein, Functions in Chloroplast Development and Abiotic Stress Responses
Source: PLoS One. 2012 Aug 8;7(8):e42924. doi: 10.1371/journal.pone.0042924 (PMC3414458; doi:10.1371/journal.pone.0042924)
Supplement: Figure S1 — Detection of MDA1 transcripts in Col-0 and mda1 mutants. (A) Structure of the At4g14605 (MDA1) gene indicating the positions of the T-DNA insertions in mda1-1 and mda1-2 by triangles. Boxes and lines indicate exons and introns, respectively. White boxes correspond to the 5′ and 3′ untranslated regions. Oligonucleotides used to study MDA1 expression are represented by horizontal arrows (not drawn to scale; Table S4). (B–C) PCR amplifications were performed using genomic DNA (gDNA) or complementary DNA (cDNA) from 2-week-old plants and primers hybridizing with (B) genomic sequences flanking the insertions in the mda1 mutants or (C) the LB of the T-DNAs and the upstream genomic region. The OTC gene was used as an internal control [56]. (PPT) [file pone.0042924.s001.ppt]

## Slide 1
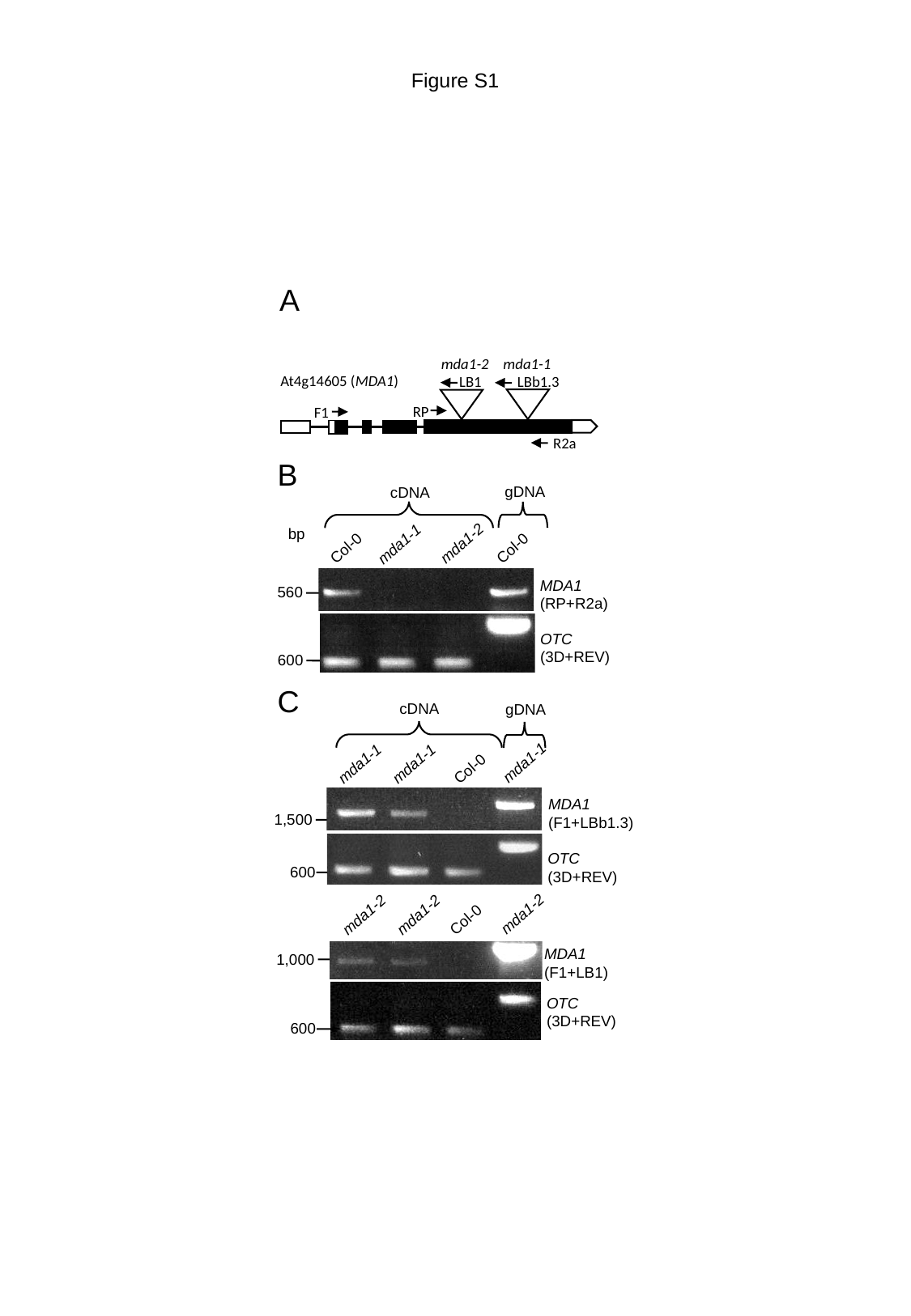

Figure S1
A
mda1-2
mda1-1
At4g14605 (MDA1)
LB1
LBb1.3
RP
F1
R2a
B
gDNA
cDNA
mda1-2
mda1-1
Col-0
Col-0
MDA1
(RP+R2a)
560
OTC
(3D+REV)
600
bp
C
cDNA
gDNA
mda1-1
mda1-1
mda1-1
Col-0
MDA1
(F1+LBb1.3)
1,500
OTC
(3D+REV)
600
mda1-2
mda1-2
mda1-2
Col-0
MDA1
(F1+LB1)
1,000
OTC
(3D+REV)
600
