# Supplementary material for: Arabidopsis MDA1, a Nuclear-Encoded Protein, Functions in Chloroplast Development and Abiotic Stress Responses
Source: PLoS One. 2012 Aug 8;7(8):e42924. doi: 10.1371/journal.pone.0042924 (PMC3414458; doi:10.1371/journal.pone.0042924)
Supplement: Figure S2 — Quantitative RT-PCR analysis of the expression of the MDA1 gene. (A) Level of expression of the MDA1 gene in the wild type (Col-0) and the mda1 mutants and (B) in different organs of Col-0 plants after normalization with those of the OTC gene (see Material and methods). Bars indicate relative levels of expression, determined as 2−ΔΔCT. A value of 1 is assigned to MDA1 expression in Col-0 (A) and roots (B). Error bars indicate the range of variation of the 2−ΔΔCT values, obtained using three different biological replicates and triplicate reactions. (PPT) [file pone.0042924.s002.ppt]

## Slide 1
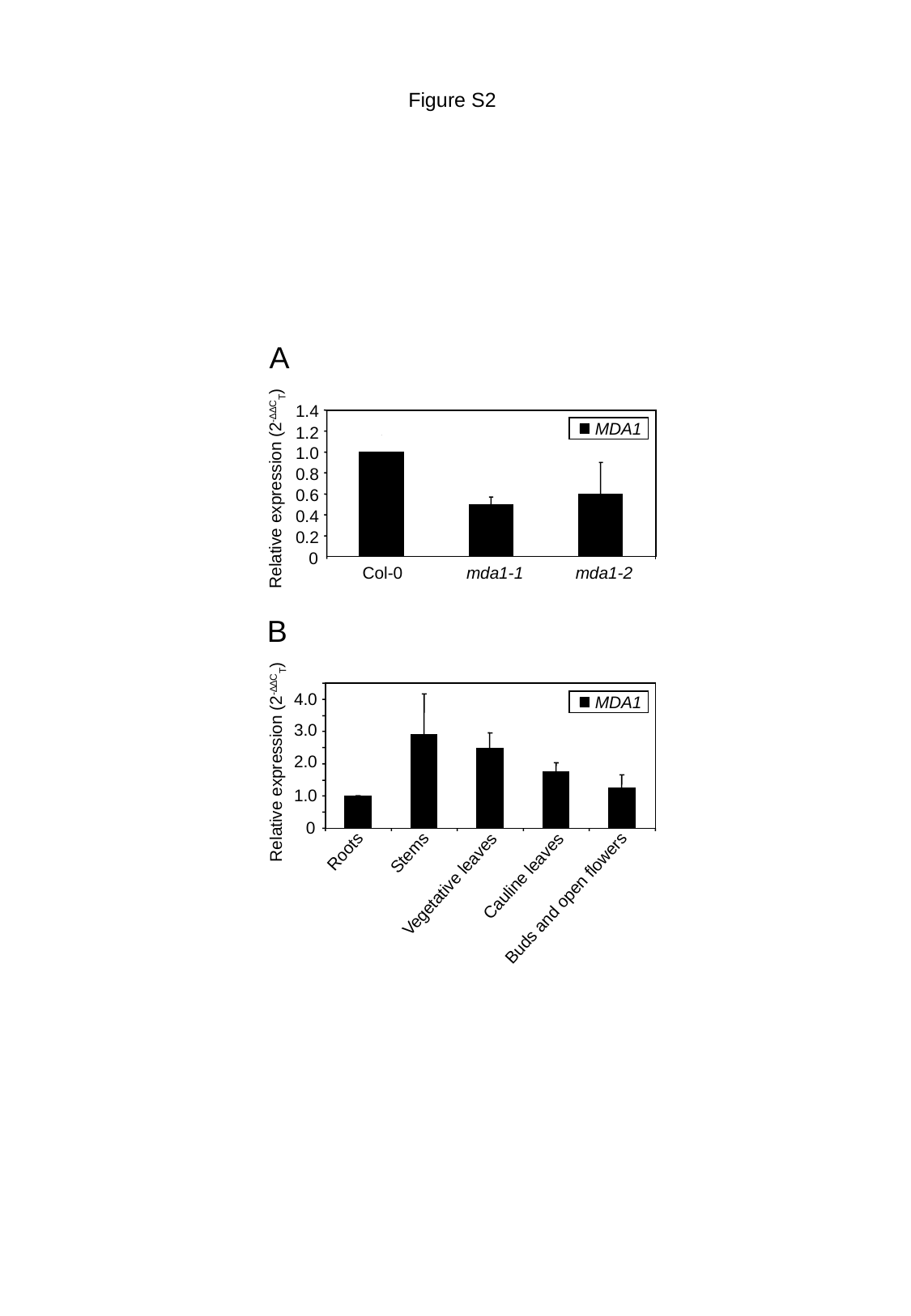

Figure S2
A
1.4
MDA1
1.2
1.0
Relative expression (2-∆∆CT)
0.8
0.6
0.4
0.2
0
Col-0
mda1-1
mda1-2
B
4.0
MDA1
3.0
Relative expression (2-∆∆CT)
2.0
1.0
0
Roots
Stems
Cauline leaves
Vegetative leaves
Buds and open flowers
