# Supplementary figures and images for: Arabidopsis MDA1, a Nuclear-Encoded Protein, Functions in Chloroplast Development and Abiotic Stress Responses
Source: PLoS One. 2012 Aug 8;7(8):e42924. doi: 10.1371/journal.pone.0042924 (PMC3414458; doi:10.1371/journal.pone.0042924)

## Slide 1
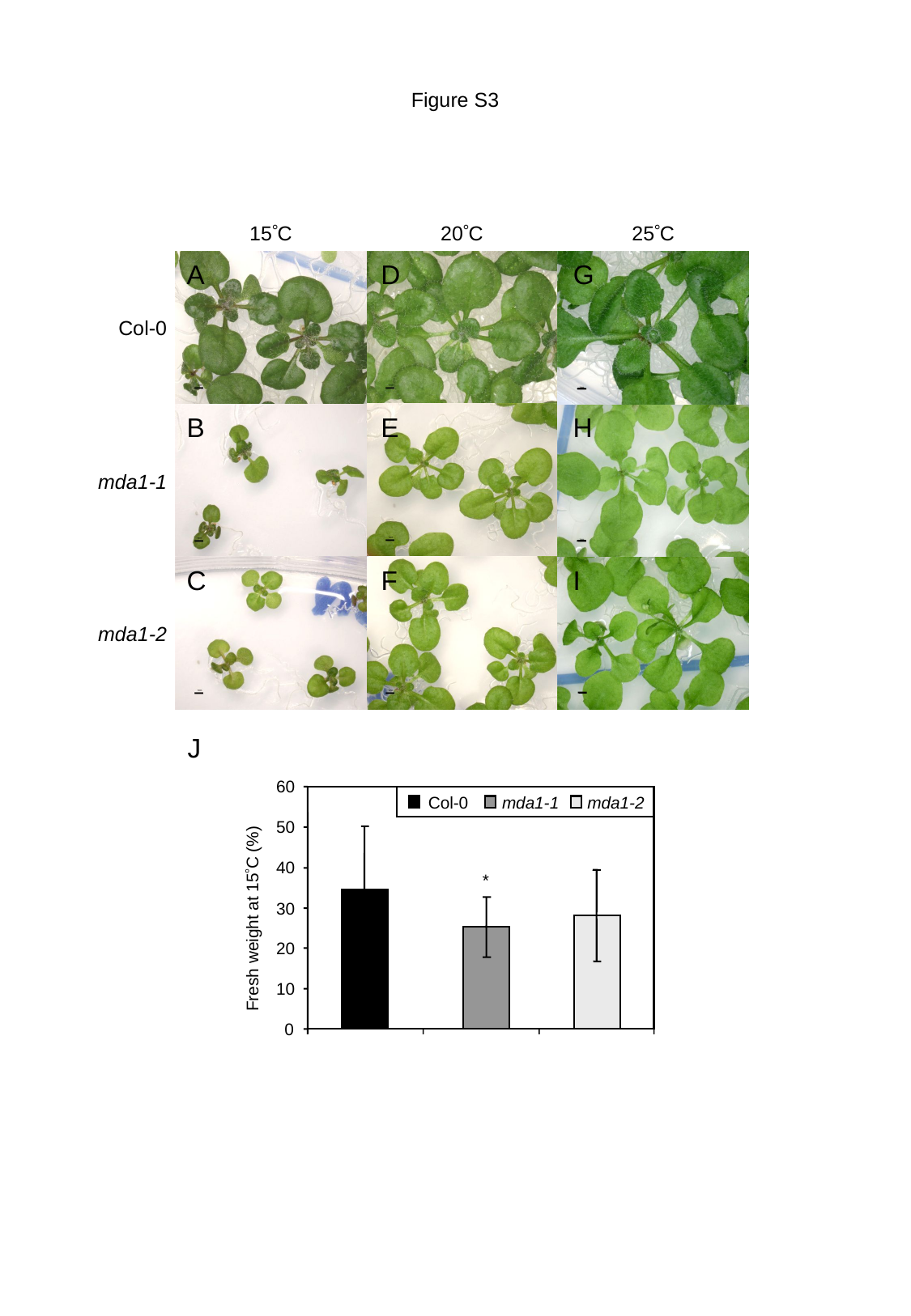

Figure S3
15°C
20°C
25°C
A
D
G
Col-0
B
E
H
mda1-1
C
F
I
mda1-2
J
60
Col-0
mda1-1
mda1-2
50
40
*
30
Fresh weight at 15°C (%)
20
10
0

Supplement: Figure S3 — Effect of temperature on the growth of the mda1 mutants. Representative plants are shown for (A, D, G) Col-0, (B, E, H) mda1-1 and (C, F, I) mda1-2, which were grown at 15 (A–C), 20 (D–F) or 25°C (G–I). (J) Fresh weight of Col-0 and mda1 individuals grown 21 days at 15°C. Each value represents the percentage of fresh weight of unstressed plants (grown at 20°C). Error bars indicate the mean ± standard deviation (SD) of the fresh weight of 15 plants of each genotype. An asterisk indicates that the value is significantly different from the wild type at P<0.05 using Student’s t-test. Pictures were taken 21 das. Scale bars indicate 1 mm. (PPT) [file pone.0042924.s003.ppt]
