# Supplementary material for: Arabidopsis MDA1, a Nuclear-Encoded Protein, Functions in Chloroplast Development and Abiotic Stress Responses
Source: PLoS One. 2012 Aug 8;7(8):e42924. doi: 10.1371/journal.pone.0042924 (PMC3414458; doi:10.1371/journal.pone.0042924)
Supplement: Figure S4 — Sugar sensitivity of the mda1-1 mutant. Pictures are shown for (A, B) Col-0 and (C, D) mda1-1 plants grown (A, C) in the presence of 30 mM or (B, D) 175 mM of sucrose. (E) Concentration (mg/g of fresh weight) of chlorophyll a (Ca) and b (Cb) in Col-0 and mda1-1 plants grown on media supplemented with 30 or 175 mM of sucrose. Data represent mean of 10 samples of 15-day-old plants per genotype ± SD. One and two asterisks indicate that the values are significantly different at P<0.05 or P<0.01, respectively, using Student’s t-test. Pictures were taken 21 das. Scale bars indicate 1 mm. (PPT) [file pone.0042924.s004.ppt]

## Slide 1
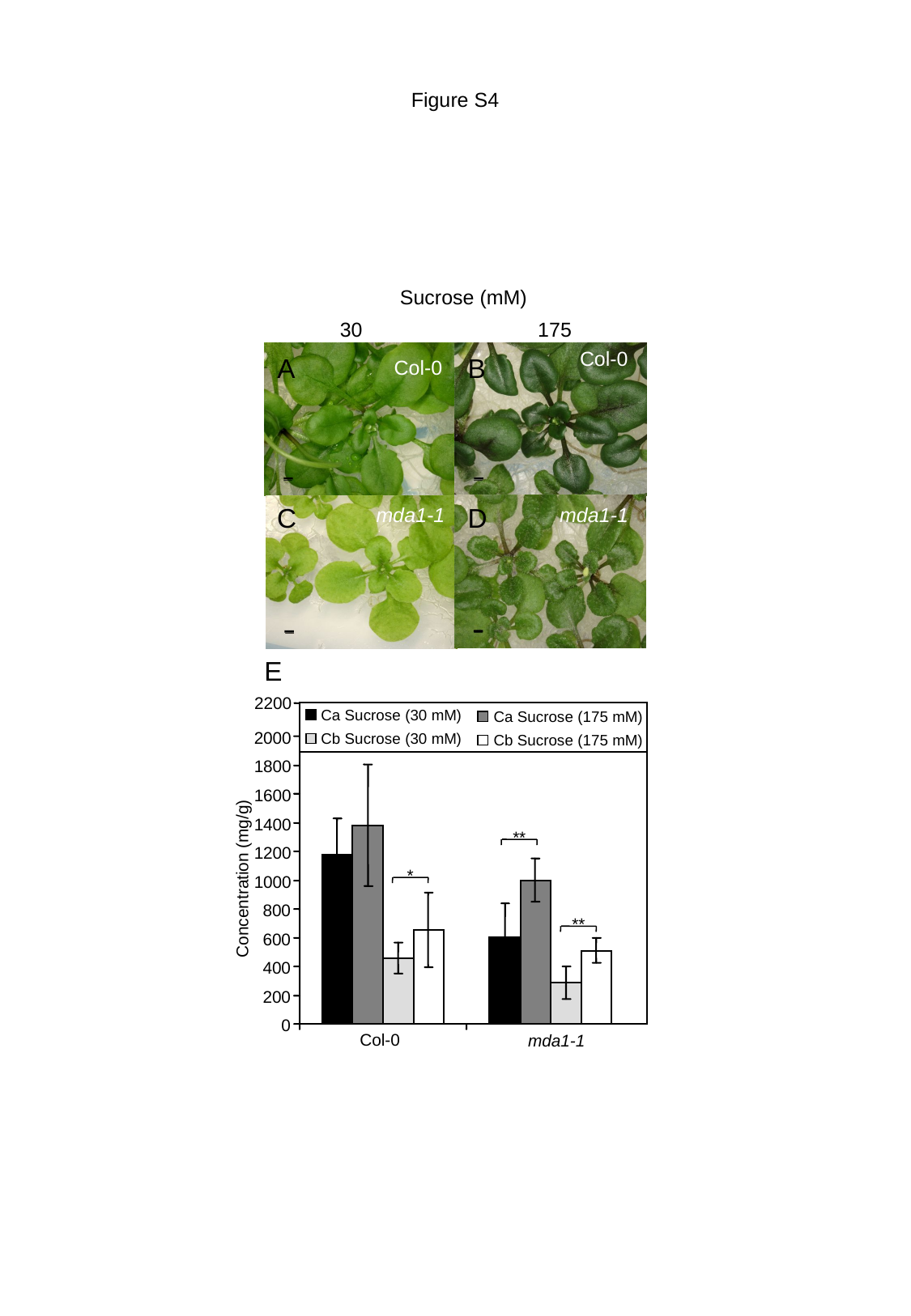

Figure S4
Sucrose (mM)
30
175
Col-0
A
B
Col-0
C
D
mda1-1
mda1-1
E
2200
Ca Sucrose (30 mM)
Ca Sucrose (175 mM)
2000
Cb Sucrose (30 mM)
Cb Sucrose (175 mM)
1800
1600
1400
**
1200
*
Concentration (mg/g)
1000
800
**
600
400
200
0
Col-0
mda1-1
