# Supplementary material for: Arabidopsis MDA1, a Nuclear-Encoded Protein, Functions in Chloroplast Development and Abiotic Stress Responses
Source: PLoS One. 2012 Aug 8;7(8):e42924. doi: 10.1371/journal.pone.0042924 (PMC3414458; doi:10.1371/journal.pone.0042924)
Supplement: Table S1 — Arabidopsis mTERF proteins. (DOC) [file pone.0042924.s005.doc]

**Table S1.** Arabidopsis mTERF proteins

| Gene  (AGI code) | CMS^a^ | Expression^b^ | Number of introns | GenBank accession number | Number of amino acid residues | Number of mTERF motifs^c^ | Prediction of the subcellular localization by TargetP v1.1^d^ | | | | |
| --- | --- | --- | --- | --- | --- | --- | --- | --- | --- | --- | --- |
|  |  |  |  |  |  |  | cTP^e^ | mTP^f^ | SP^g^ | Other | Localization^h^ |
| AT1G21150 | I | ECM | 0 | NM_101969 | 390 | 5 | 0.302 | 0.213 | 0.039 | 0.093 | C |
| AT1G56380 | I | N.D. | 1 | NM_104517 | 388 | 7 | 0.106 | 0.075 | 0.06 | 0.901 | - |
| AT1G61960 | I | EC | 0 | NM_104876 | 457 | 7 | 0.023 | 0.928 | 0.011 | 0.047 | Mt |
| AT1G61970 | I | ECM | 1 | NM_104877 | 418 | 6 | 0.172 | 0.819 | 0.002 | 0.014 | Mt |
| AT1G61980 | I | ECM | 0 | NM_104878 | 418 | 6 | 0.304 | 0.869 | 0.001 | 0.019 | Mt |
| AT1G61990 | I | EC | 0 | NM_104879 | 414 | 5 | 0.049 | 0.766 | 0.096 | 0.008 | Mt |
| AT1G62010 | I | ECM | 0 | NM_104881 | 415 | 6 | 0.019 | 0.807 | 0.051 | 0.024 | Mt |
| AT1G62085 | I | EM | 0 | NM_202340 | 461 | 7 | 0.088 | 0.78 | 0.007 | 0.092 | Mt |
| AT1G62110 | I | ECM | 0 | NM_104892 | 462 | 7 | 0.017 | 0.969 | 0.004 | 0.038 | Mt |
| AT1G62120 | I | ECM | 0 | NM_104893 | 437 | 6 | 0.018 | 0.933 | 0.004 | 0.045 | Mt |
| AT1G62150 | I | ECM | 0 | NM_104896 | 463 | 7 | 0.225 | 0.524 | 0.005 | 0.184 | Mt |
| AT1G62490 | I | E | 1 | NM_104928 | 334 | 3 | 0.082 | 0.136 | 0.01 | 0.686 | - |
| AT1G74120 | I | EM | 0 | NM_106072 | 445 | 5 | 0.111 | 0.274 | 0.006 | 0.226 | Mt |
| AT1G78930 | I | EC | 6 | NM_106542 | 591 | 8 | 0.861 | 0.034 | 0.015 | 0.135 | C |
| AT1G79220 | I | ECM | 0 | NM_106573 | 399 | 6 | 0.013 | 0.791 | 0.468 | 0.004 | Mt |
| AT2G03050 | II | ECM | 0 | NM_126357 | 283 | 5 | 0.204 | 0.206 | 0.02 | 0.173 | Mt |
| AT2G21710 | II | ECM | 5 | NM_127741 | 641 | 8 | 0.183 | 0.213 | 0.044 | 0.225 | - |
| AT2G34620 | II | ECM | 1 | NM_129016 | 303 | 6 | 0.706 | 0.071 | 0.025 | 0.139 | C |
| AT2G36000 | II | ECM | 1 | NM_179927 | 318 | 5 | 0.945 | 0.042 | 0.015 | 0.023 | C |
| AT2G44020 | II | ECM | 0 | NM_129964 | 507 | 9 | 0.026 | 0.657 | 0.077 | 0.034 | Mt |
| AT3G18870 | III | ECM | 0 | NM_112773 | 275 | 5 | 0.974 | 0.045 | 0.009 | 0.040 | C |
| AT3G46950 | III | ECM | 0 | NM_114562 | 450 | 7 | 0.074 | 0.792 | 0.008 | 0.089 | Mt |
| AT3G60400 | III | EM | 0 | NM_115904 | 558 | 5 | 0.167 | 0.545 | 0.045 | 0.032 | Mt |
| AT4G02990 | IV | ECM | 1 | NM_116533 | 541 | 10 | 0.191 | 0.264 | 0.004 | 0.098 | Mt |
| AT4G09620 | IV | ECM | 2 | NM_117030 | 212 | 0 | 0.409 | 0.09 | 0.071 | 0.171 | C |
| AT4G14605 | IV | ECM | 4 | NM_117541 | 444 | 7 | 0.438 | 0.316 | 0.008 | 0.245 | C |
| AT4G19650 | IV | EM | 3 | NM_118085 | 575 | 6 | 0.248 | 0.075 | 0.007 | 0.919 | - |
| AT4G38160 | IV | ECM | 1 | NM_202974 | 363 | 7 | 0.037 | 0.089 | 0.058 | 0.934 | - |
| AT5G06810 | V | EM | 1 | NM_120764 | 1141 | 8 | 0.234 | 0.738 | 0.018 | 0.025 | Mt |
| AT5G07900 | V | ECM | 0 | NM_120872 | 405 | 5 | 0.490 | 0.106 | 0.038 | 0.075 | C |
| AT5G23930 | V | EM | 0 | NM_122298 | 457 | 7 | 0.082 | 0.910 | 0.009 | 0.096 | Mt |
| AT5G45113 | V | E | 0 | NM_148090 | 414 | 0 | 0.027 | 0.237 | 0.115 | 0.810 | - |
| AT5G55580 | V | ECM | 4 | NM_124940 | 496 | 6 | 0.367 | 0.121 | 0.121 | 0.100 | C |
| AT5G64950 | V | ECM | 0 | NM_125894 | 391 | 7 | 0.192 | 0.865 | 0.005 | 0.045 | Mt |
| AT5G54180 | V | ECM | 1 | NM_124798 | 500 | 8 | 0.889 | 0.018 | 0.137 | 0.005 | C |

^a^CMS: Chromosome. ^b^Gene expression evidence obtained from EST (E), cDNA (A) and MPSS (M; *massive parallel signature sequencing*). ^c^ mTERF motifs identified by the SMART program (http://smart.embl-heidelberg.de/). ^d^Subcellular localization predicted by the TargetP v1.1 tool (http://www.cbs.dtu.dk/services/TargetP/). ^e^cTP: transit peptide to chloroplasts. ^f^mTP: transit peptide to mitochondria. ^g^SP: Secreted protein. ^h^Subcellular localization: Mt (Mitochondrial); C (Chloroplastic); - (unknown). N.D. not detected. Similar results were obtained when using the PREDOTAR V1.03 (http://urgi.versailles.inra.fr/predotar/predotar.html; [17]), IPSORT (http://hc.ims.u-tokyo.ac.jp/iPSORT/; [18]) and ProteinProwler (http://pprowler.itee.uq.edu.au/pprowler_webapp_1-2/) subcellular localization tools.
