# Supplementary material for: Arabidopsis MDA1, a Nuclear-Encoded Protein, Functions in Chloroplast Development and Abiotic Stress Responses
Source: PLoS One. 2012 Aug 8;7(8):e42924. doi: 10.1371/journal.pone.0042924 (PMC3414458; doi:10.1371/journal.pone.0042924)
Supplement: Table S2 — Rice mTERF proteins. (DOC) [file pone.0042924.s006.doc]

**Table S2.** Rice mTERF proteins

| Gene^a^ | CMS^b^ | Expression^c^ | Number of introns | GenBank accession number | Number of amino acid residues | Number of mTERF motifs^d^ | Prediction of the subcellular localization by TargetP v1.1^e^ | | | | |
| --- | --- | --- | --- | --- | --- | --- | --- | --- | --- | --- | --- |
|  |  |  |  |  |  |  | cTP^f^ | mTP^g^ | SP^h^ | Other | Localization^i^ |
| LOC_Os02g39040 | II | EM | 4 | NM_001053892 | 270 | 7 | 0.006 | 0.344 | 0.173 | 0.757 | - |
| LOC_Os02g51450 | II | EM | 1 | NM_001054663 | 167 | 2 | 0.013 | 0.501 | 0.02 | 0.621 | - |
| LOC_Os02g51460 | II | EM | 0 | NM_001054664 | 381 | 5 | 0.045 | 0.766 | 0.071 | 0.008 | Mt |
| LOC_Os02g54200 | II | EM | 3 | NM_001054858 | 484 | 9 | 0.07 | 0.849 | 0.005 | 0.084 | Mt |
| LOC_Os03g24590 | III | EM | 0 | NM_001056690 | 300 | 5 | 0.222 | 0.845 | 0.003 | 0.055 | Mt |
| LOC_Os03g57149 | III | E | 0 | NM_001058016 | 309 | 6 | 0.661 | 0.205 | 0.012 | 0.081 | C |
| LOC_Os04g54510 | IV | EM | 0 | NM_001060560 | 392 | 5 | 0.218 | 0.510 | 0.002 | 0.039 | Mt |
| LOC_Os05g33440 | V | EM | 0 | NM_001062033 | 392 | 5 | 0.018 | 0.832 | 0.003 | 0.031 | Mt |
| LOC_Os05g33460 | V | EM | 0 | NM_001062034 | 398 | 6 | - | 0.958 | 0.011 | 0.089 | Mt |
| LOC_Os05g33500 | V | EM | 0 | NM_001062035 | 507 | 9 | 0.942 | 0.184 | 0.005 | 0.013 | C |
| LOC_Os05g34160 | V | EM | 0 | NM_001062079 | 394 | 4 | 0.001 | 0.687 | 0.139 | 0.010 | Mt |
| LOC_Os06g12040 | VI | EM | 0 | NM_001063723 | 391 | 5 | 0.111 | 0.741 | 0.010 | 0.038 | Mt |
| LOC_Os06g12060 | VI | E | 2 | NM_001063724 | 1007 | 9 | 0.061 | 0.837 | 0.010 | 0.031 | Mt |
| LOC_Os06g12070 | VI | EM | 2 | NM_001063726 | 411 | 4 | 0.128 | 0.747 | 0.009 | 0.049 | Mt |
| LOC_Os06g12080 | VI | EM | 0 | NM_001063727 | 391 | 4 | 0.641 | 0.225 | 0.008 | 0.073 | C |
| LOC_Os06g12100 | VI | EM | 0 | NM_001063729 | 438 | 4 | 0.908 | 0.022 | 0.001 | 0.261 | C |
| LOC_Os06g12110 | VI | EM | 0 | NM_001063730 | 392 | 4 | 0.045 | 0.847 | 0.015 | 0.053 | Mt |
| LOC_Os07g04230 | VII | EM | 5 | NM_001065379 | 484 | 8 | 0,55 | 0.695 | 0.004 | 0.025 | Mt |
| LOC_Os07g22670 | VII | EM | 0 | NM_001065976 | 574 | 5 | 0.053 | 0.916 | 0.003 | 0.013 | Mt |
| LOC_Os07g24090 | VII | EM | 0 | NM_001066021 | 407 | 5 | 0.098 | 0.680 | 0.004 | 0.050 | Mt |
| LOC_Os07g39430 | VII | EM | 5 | NM_001066649 | 502 | 7 | 0.862 | 0.578 | 0.008 | 0.020 | C |
| LOC_Os08g40430 | VIII | EM | 1 | NM_001068770 | 332 | 7 | 0.273 | 0.128 | 0.058 | 0.733 | - |
| LOC_Os08g40630 | VIII | EM | 0 | NM_001068785 | 635 | 4 | 0.425 | 0.604 | 0.002 | 0.035 | Mt |
| LOC_Os09g38720 | IX | E | 7 | NM_001070458 | 516 | 8 | 0.937 | 0.029 | 0.025 | 0.028 | C |
| LOC_Os11g09990 | XI | E | 0 | NM_001073989 | 405 | 4 | 0.102 | 0.950 | 0.001 | 0.018 | Mt |
| LOC_Os11g10000 | XI | EM | 0 | NM_001073990 | 457 | 6 | 0.004 | 0.978 | 0.009 | 0.065 | Mt |
| LOC_Os11g10040 | XI | EM | 1 | NM_001073992 | 391 | 4 | 0.081 | 0.885 | 0.055 | 0.004 | Mt |
| LOC_Os11g14130 | XI | EM | 1 | NM_001074137 | 416 | 5 | 0.300 | 0.274 | 0.003 | 0.039 | C |
| LOC_Os12g30610 | XII | EM | 1 | NM_001073330 | 364 | 2 | 0.138 | 0.891 | 0.001 | 0.027 | Mt |

^a^Locus identifier according to the Rice Genome Annotation Project (http://rice.plantbiology.msu.edu/index.shtml).  ^b^CMS: Chromosome. ^c^Gene expression evidence obtained from EST (E) and MPSS (M; *massive parallel signature sequencing*). ^d^mTERF motifs identified by the SMART program (http://smart.embl-heidelberg.de/). ^e^Subcellular localization predicted by the TargetP v1.1 tool (http://www.cbs.dtu.dk/services/TargetP/). ^f^cTP: transit peptide to chloroplasts. ^g^mTP: transit peptide to mitochondria. ^h^SP: Secreted protein. Subcellular localization: Mt (Mitochondrial); C (Chloroplastic); - (unknown). N.D. not detected. Similar results were obtained when using the PREDOTAR V1.03 (http://urgi.versailles.inra.fr/predotar/predotar.html; [17]), IPSORT (http://hc.ims.u-tokyo.ac.jp/iPSORT/; [18]) and ProteinProwler (http://pprowler.itee.uq.edu.au/pprowler_webapp_1-2/) subcellular localization tools.
