# Supplementary material for: Arabidopsis MDA1, a Nuclear-Encoded Protein, Functions in Chloroplast Development and Abiotic Stress Responses
Source: PLoS One. 2012 Aug 8;7(8):e42924. doi: 10.1371/journal.pone.0042924 (PMC3414458; doi:10.1371/journal.pone.0042924)
Supplement: Table S3 — T-DNA lines for mTERF genes used in this work. (DOC) [file pone.0042924.s007.doc]

**Table S3.** T-DNA lines for the *mTERF* genes studied in this work

| Line | Gene (AGI code) |
| --- | --- |
| N840727 | At1g21150 |
| N858700 | At1g21150 |
| N574151 | At1g56360 |
| N550757 | At1g56380 |
| N550758 | At1g56380 |
| N550766 | At1g56380 |
| N585679 | At1g56380 |
| N627489 | At1g56380 |
| N809767 | At1g61960 |
| N596241 | At1g61980 |
| N627339 | At1g61980 |
| N856562 | At1g62010 |
| N821972 | At1g62085 |
| N828440 | At1g62085 |
| N821971 | At1g62085 |
| N616402 | At1g62085 |
| N522812 | At1g62110 |
| N543629 | At1g62110 |
| N644284 | At1g62110 |
| N510348 | At1g62120 |
| N552797 | At1g62120 |
| N826458 | At1g62150 |
| N848714 | At1g62160 |
| N524146 | At1g62490 |
| N526295 | At1g62490 |
| N574127 | At1g62490 |
| N526816 | At1g74120 |
| N634099 | At1g74120 |
| N640329 | At1g74120 |
| N640332 | At1g74120 |
| N515344 | At1g78930 |
| N614981 | At1g78930 |
| N536989 | At1g79220 |
| N574559 | At1g79220 |
| N517427 | At2g21710 |
| N538426 | At2g21710 |
| N538435 | At2g21710 |
| N641541 | At2g21710 |
| N597699 | At2g34620 |
| N800536 | At2g34620 |
| N597691 | At2g34620 |
| N586466 | At2g44020 |
| N851737 | At2g44020 |
| N810199 | At3g18870 |
| N856600 | At3g18870 |
| N851820 | At3g46950 |
| N842389 | At3g46950 |
| N612094 | At3g46950 |
| N641630 | At3g60400 |
| N803866 | At3g60400 |
| N878707 | At4g09620 |
| N597243 | At4g14605 |
| N808202 | At4g14605 |
| N819625 | At4g14605 |
| N513581 | At4g19650 |
| N622281 | At4g19650 |
| N817217 | At4g19650 |
| N837231 | At4g19650 |
| N549512 | At5g06810 |
| N572208 | At5g06810 |
| N821565 | At5g06810 |
| N561296 | At5g07900 |
| N847740 | At5g07900 |
| N512266 | At5g45113 |
| N541080 | At5g45113 |
| N541081 | At5g45113 |
| N824947 | At5g45113 |
| N627626 | At5g54180 |
| N810368 | At5g54180 |
| N620765 | At5g55580 |
| N643391 | At5g55580 |
| N651197 | At5g55580 |
| N651198 | At5g55580 |
| N857510 | At5g55580 |
| N844970 | At5g64950 |
