# Supplementary material for: Arabidopsis MDA1, a Nuclear-Encoded Protein, Functions in Chloroplast Development and Abiotic Stress Responses
Source: PLoS One. 2012 Aug 8;7(8):e42924. doi: 10.1371/journal.pone.0042924 (PMC3414458; doi:10.1371/journal.pone.0042924)
Supplement: Table S4 — Primers used in this work. (DOC) [file pone.0042924.s008.doc]

**Table S4. Primers used in this work**

| Purpose | Primer names  (forward/reverse) | Oligonucleotide sequences (5´→3´) | |
| --- | --- | --- | --- |
|  |  | Forward primer | Reverse primer |
| RT-PCR | At4g14605 F1/R1 | GTCAACTTGGACCTTCAGAG | TGCAGCAGAATCTGTGTCAC |
|  | At4g14605 R2a |  | CCAGCTCATATCTCGGCTTG |
|  | OTC3D/R | TCCTTGCCAAATCATGGCCG | GCATGCATGCGATTCTCCGC |
| Confirmation of T-DNA insertions | LBa1 |  | TGGTTCACGTAGTGGGCCATCG |
|  | LB | AGTTGCAGCAAGCGGTCCACGC |  |
|  | LB3 | GTGTATCGAGATTGGTTATGAAATTCAGATGCTA |  |
|  | LB1 |  | GCCTTTTCAGAAATGGATAAATAGCCTTGCTTCC |
|  | LBb1.3 | ATTTTGCCGATTTCGGAAC |  |
|  | LB-pDs-Lox | GTCCGCAATGTGTTATTAAGTTG |  |
|  | At4g14605 LP/RP | TGTTGAATGGGAAGACAAAGC | AACCGATAACCTGAAACCGAC |
|  | At2g03050 F/R | CAAAGCTCTCCGAGTAAACCT | GATTTAGGTATGTCTTGCTCTG |
|  | At4g02990 F3/R4 | AGGTTCTGGCTTTGAACCTC | CCTCTCATGATGATGATACAT |
| qRT-PCR | At4g14605 F/R(Q) | TACCTCGTAGGAAGAGAGCTTA | GGATCAGGGAAGCTCACAACA |
|  | At2g24120 F/R(Q) | CTTGGTGATTGTGCAAAGATAATT | GGGAGGAAATGCAGTTCTTTGTT |
|  | AtCg00020 F/R(Q) | TGCTGCTCCTCCAGTAGATATT | CAATAGCTGCAGAAGTAGGAATA |
|  | AtCg00040 F/R(Q) | TGTATGAACAGAATCGTTTGATTAT | AATTTCGACAATCACTGCAAATA |
|  | AtCg00190 F/R(Q) | GCAGGTTAGAATTAGAGATTGATA | GGGTAGCAAACATTCTCTAGAAT |
|  | AtCg00500 F/R (Q) | GCTAAGTAAAGCAATGGATAGTTT | CGAATGTCCTTGGAGCTAACTAA |
|  | AtCg00650 F/R (Q) | GACGGGTGAATAGAGTGACTTT | GGAGTCGACTCACTTCTTTCAA |
|  | AtCg00670 F/R (Q) | GGTTGACATATACAACCGACTTT | CCATCCACCAGGAGAGTTTATA |
|  | OTC F/R(Q) | TGAAGGGACAAAGGTTGTGTA | CGCAGACAAAGTGGAATGGA |
