# Supplementary material for: Arabidopsis MDA1, a Nuclear-Encoded Protein, Functions in Chloroplast Development and Abiotic Stress Responses
Source: PLoS One. 2012 Aug 8;7(8):e42924. doi: 10.1371/journal.pone.0042924 (PMC3414458; doi:10.1371/journal.pone.0042924)
Supplement: Table S5 — Tiling array expression for Arabidopsis mTERF genes. (DOC) [file pone.0042924.s009.doc]

**Table S5.** Tiling array expression for Arabidopsis *mTERF* genes

| Gene  (AGI code) | Before treatment | Treatment (1 hour) | | | | Treatment (12 hours) | | | |
| --- | --- | --- | --- | --- | --- | --- | --- | --- | --- |
|  |  | Mock | NaCl (200 mM) | Mannitol (300 mM) | ABA (100 μM) | Mock | NaCl (200 mM) | Mannitol (300 mM) | ABA (100 μM) |
| AT1G21150 | 1.2718 | 0.9346 | 0.9603 | 0.7797 | 1.0250 | 1.4698 | 0.6228 | 0.9380 | 0.9534 |
| AT1G56380 | 0.9887 | 0.9781 | 0.9214 | 1.0193 | 1.2235 | 0.9465 | 1.0590 | 0.9298 | 1.1119 |
| AT1G61960 | 1.0642 | 1.0618 | 0.9188 | 0.9390 | 0.7263 | 1.3302 | 0.9559 | 1.0736 | 0.7954 |
| AT1G61970 | 1.1420 | 1.0791 | 1.0047 | 1.0263 | 0.7870 | 1.1651 | 0.8394 | 0.9228 | 0.8918 |
| AT1G61980 | 0.9927 | 0.9875 | 0.7805 | 0.9519 | 0.7819 | 1.3125 | 1.0827 | 1.1610 | 0.8765 |
| AT1G61990 | 0.9424 | 0.9382 | 0.6818 | 0.8813 | 0.7209 | 1.5093 | 0.9482 | 1.0746 | 0.8582 |
| AT1G62010 | 0.8490 | 1.1081 | 0.6636 | 0.9269 | 0.7606 | 1.4393 | 0.9892 | 0.9006 | 0.6710 |
| AT1G62085 | 0.9852 | 1.0459 | 0.9705 | 0.9687 | 1.0084 | 1.1282 | 0.9100 | 0.8415 | 0.9600 |
| AT1G62110 | 0.7744 | 0.9376 | 0.7279 | 0.7634 | 0.8494 | 1.6588 | 0.9932 | 1.1616 | 0.9784 |
| AT1G62120 | 1.0800 | 1.1303 | 0.9256 | 0.9095 | 1.0260 | 1.2137 | 0.6497 | 0.9110 | 0.9394 |
| AT1G62150 | 0.6878 | 0.8896 | 0.6782 | 0.7026 | 0.7680 | 1.4922 | 1.0671 | 0.9943 | 0.8152 |
| AT1G62490 | 0.8381 | 0.7964 | 0.9493 | 1.0998 | 1.1047 | 0.9867 | 0.9194 | 0.9873 | 1.0048 |
| AT1G74120 | 1.1781 | 1.0474 | 1.1065 | 0.9028 | 0.8627 | 1.1479 | 0.8779 | 0.9180 | 0.9904 |
| AT1G78930 | 0.5347 | 0.5373 | 0.4727 | 0.4765 | 0.4526 | 1.2314 | 1.1101 | 0.8749 | 0.7448 |
| AT1G79220 | 1.2239 | 0.9461 | 0.8088 | 0.8746 | 0.6921 | 1.2971 | 0.8155 | 1.0113 | 0.7785 |
| AT2G03050^a^ | 1.9632 | 0.8908 | 1.0410 | 0.9424 | 0.8712 | 1.1451 | 0.7801 | 0.7124 | 0.8710 |
| AT2G21710 | 1.0183 | 0.8246 | 0.7658 | 0.8199 | 0.6164 | 1.3658 | 0.9637 | 1.0069 | 0.8189 |
| AT2G34620 | 3.2143 | 0.3052 | 1.0325 | 1.1357 | 0.6623 | 0.8285 | 0.3980 | 0.3144 | 0.5996 |
| AT2G36000 | 1.4637 | 0.6970 | 0.7380 | 0.8464 | 0.6834 | 1.0775 | 0.5112 | 0.8312 | 0.8486 |
| AT2G44020 | 0.8773 | 1.2649 | 0.9716 | 0.8888 | 1.0320 | 1.1759 | 0.9552 | 1.0142 | 0.8555 |
| AT3G18870 | 1.5649 | 0.8205 | 0.9314 | 0.7233 | 0.6780 | 1.2248 | 0.5252 | 0.6371 | 0.7126 |
| AT3G46950 | 0.7616 | 0.9223 | 0.5753 | 0.8430 | 0.9623 | 1.3072 | 1.1795 | 1.3500 | 0.9804 |
| AT3G60400 | 1.1061 | 1.1866 | 1.0575 | 1.0540 | 0.8968 | 1.1047 | 0.9595 | 0.8328 | 0.8394 |
| AT4G02990^b^ | 0.8961 | 0.7883 | 0.6915 | 0.6918 | 0.6093 | 1.5071 | 0.8131 | 1.0944 | 0.8740 |
| AT4G09620 | 1.3913 | 1.1173 | 1.1399 | 1.0754 | 0.9466 | 1.0209 | 0.7060 | 0.9864 | 0.9486 |
| AT4G14605^c^ | 1.5801 | 0.9678 | 1.0117 | 1.2257 | 0.7197 | 1.0363 | 0.6726 | 0.8646 | 0.6686 |
| AT4G19650 | 0.9464 | 1.0349 | 0.9881 | 0.8495 | 0.7964 | 1.1050 | 1.0566 | 0.8709 | 0.8725 |
| AT4G38160 | 1.5755 | 0.8074 | 0.8955 | 1.0421 | 0.6765 | 1.5726 | 0.4777 | 1.7157 | 0.7113 |
| AT5G06810 | 0.8552 | 1.1928 | 0.7705 | 0.9021 | 0.8174 | 1.3239 | 0.9142 | 1.0687 | 0.8328 |
| AT5G07900 | 0.9783 | 0.8358 | 0.7338 | 0.8431 | 0.7129 | 1.6321 | 1.0160 | 1.1011 | 0.7524 |
| AT5G23930 | 0.6077 | 0.8600 | 0.6487 | 0.7198 | 0.7818 | 1.5802 | 1.3828 | 1.4318 | 1.1529 |
| AT5G45113 | 0.9011 | 0.8615 | 0.9856 | 1.0065 | 1.1948 | 0.8677 | 1.1157 | 0.9866 | 1.1996 |
| AT5G55580 | 1.1667 | 0.8862 | 0.7185 | 0.7244 | 0.5948 | 1.5992 | 1.1817 | 1.0928 | 0.7173 |
| AT5G64950 | 0.9527 | 1.1539 | 0.7680 | 0.9832 | 0.8439 | 1.2681 | 0.8560 | 0.9642 | 0.8329 |
| AT5G54180 | 1.2486 | 1.0233 | 1.1567 | 1.2464 | 0.8229 | 1.3839 | 0.7094 | 0.9413 | 0.7665 |

Intensity mean-normalized values for the expression of Arabidopsis *mTERF* genes. Data were obtained from whole-genome tiling array hybridization experiments performed using RNA extracted from 10 day-old Col-0 seedlings under different abiotic stress conditions [23]. The information is available at the *Arabidopsis thaliana* tiling Array Express (At-TAX) homepage (http://www.weigelworld.org/resources/microarray/at-tax) and can be visualized using the TileViz visualization tool (http://jsp.weigelworld.org/tileviz/tileviz.jsp). ^a^*SOLDAT10*, ^b^*RUG2* and ^c^*MDA1* genes.
